# The First Record and Classification of Planktonic Radiolarian (*Phylum Retaria*) and Phaeodarian (*Phylum Cercozoa*) in the Eastern Indian Ocean

**DOI:** 10.3390/biology10030202

**Published:** 2021-03-08

**Authors:** Sonia Munir, Jun Sun, Steve L. Morton

**Affiliations:** 1 Research Centre for Indian Ocean Ecosystem, Tianjin University of Science and Technology, Tianjin 300457, China; soniaku2003@yahoo.com; 2 College of Marine Science and Technology, China University of Geosciences, Wuhan 430074, China; 3 National Ocean Service, NOAA, 331 Fort Johnson Road, Charleston, SC 29412, USA; steve.morton@noaa.gov

**Keywords:** planktonic Radiolarian, *Phylum Retaria*, *Phylum Cercozoa*, Acanthria, Collodaria, Pheodaria, Taxopodida, Polycystinea, eastern Indian Ocean

## Abstract

**Simple Summary:**

*Phylum Retaria* and *Phylum Cercozoa* consists of the siliceous planktonic organisms, commonly referred to as Radiolarians, were investigated from 200 m depth to the surface in the eastern Indian Ocean (80.00°–96.10° E, 10.08° N–6.00° S) during a 2 months cruise (10 April–13 May 2014). Samples collected from 44 locations were analyzed by using both light and electron microscopy. Out of 168 taxa, 60 newly recorded species from the groups i.e., Acantharia, Collodaria, Pheodaria, Taxopodida and Polycystinea were recorded for the first time.

**Abstract:**

Siliceous planktonic species of the phyla Retaria and Cercozoa were investigated from the surface to a 200 m depth around the eastern Indian Ocean (80.00°–96.10° E, 10.08° N–6.00° S) during a 2-month cruise (10 April–13 May 2014). These species are commonly referred to as Radiolarians and are found in all of the world’s oceans; however, this is a detailed investigation of the species’ diversity in the eastern Indian Ocean. Samples were collected from the eastern Indian Ocean using a plankton towing net during a vertical haul from 44 sampling stations, which resulted in 168 taxa, including 60 species that were newly recorded in the study area. The main purpose of this work was to identify members of the phyla Retaria and Cercozoa and their distribution in the eastern Indian Ocean. The species’ morphology, identification, notes, and new geographical records are briefly described.

## 1. Introduction

The eastern Indian Ocean (EIO) is a typical oligotrophic region and is well known for its monsoon circulation, water stratification, and equatorial Wyrtki Jet current [1]. This particular set of circumstances make it a global biogeochemical and ecological hotspot for microplankton species in an open-sea research area [2].

Planktonic Radiolarians are silicate skeletal shell organisms which are found at every depth from the surface to the deepest part of the ocean [3]. Siliceous plankton are potentially grazers, which have an important role in the tropical food chain and increase the amounts of carbon and nutrients such as silica in the ocean [4]. The opaline silicate shells of Radiolarians remain in ocean sediments and form a large siliceous ooze. The mineralized shells found in sediment samples have been widely used in evolution, biology, and paleontology research [5].

The phyla Retaria and Cercozoa are two groups of Radiolaria and are divided into four classes—Acantharia, Taxopodia, Polycystine, and Pheodaria [6,7,8]. Celestine-based and siliceous-shelled organisms are included in these groups and can be characterized by an organic-walled, rather simple, central capsule surrounded by long tapered protoplasmic axopodial spines [9,10]. More than 2500 genera and 15,000 species have been described in the World Ocean [11]. Initially, in 1887 Haeckel reported higher taxa of Radiolaria from sediment samples taken during expeditions around the Pacific Ocean, Atlantic Ocean, Arctic Ocean, and Indian Ocean [6,12]. Recently, our knowledge of many species has been revised on the basis of morphological and molecular variation, causing changes in the classification of Radiolarians. These revisions in the group of Radiolarians incorporate recent advances and provide new insight into the taxonomic group of Radiolarians that contradicts the older artificial Haeckel 1887 classification [9,13]. The relatively limited and incomplete classification of these planktonic species offers substantial opportunity to conduct more taxonomic and systematic research, as exemplified by some recent evolutionary studies [9]. Several groups of Radiolarians are structurally similar—i.e., Acantharia and Taxopodia; it is still debated whether they belong to the Polycystine group [14,15]. Despite these debated issues, these two groups now have been separated into different classes on the basis of molecular studies [15], which are still discovering novel organisms [9].

The main objective of this study was to identify members of the phyla Retaria and Cercozoa and predict their distribution prospects in the eastern Indian Ocean. Data resources for the Radiolarian community are limited in the eastern Indian Ocean; only a few articles have been published by Johnson and Niringi [16]. Their findings were based solely on the Polycystinea group, members of which were collected from the surface sediments of the eastern Indian Ocean. Many taxa still need to be explored because of the scarcity of work and reports from this region. We conducted a detailed investigation on planktonic Radiolarians collected by plankton net tow sampling during Shiyan 1 cruises in the spring period of 2014. This taxonomic report on newly recorded Radiolarian taxa in the eastern Indian Ocean will be a major contribution to the scientific community and further our knowledge on the distribution of Radiolarians.

## 2. Materials and Methods

### 2.1. Identification from Plankton Net Tow Samples

**Figure 1:**
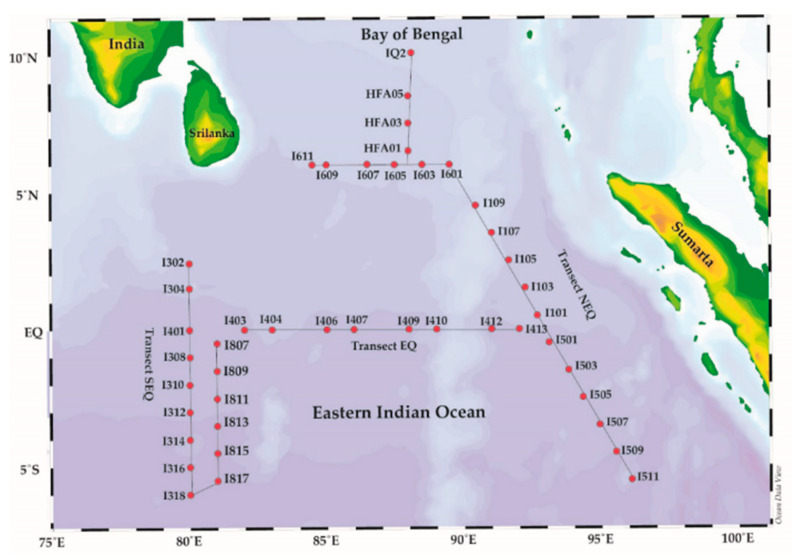
Sampling stations of the eastern Indian Ocean (EIO) during the spring period, 2014. The red dots indicate sampling stations between the three regional zones: North-equatorial zone (NEQ) at longitude 90° E, the equatorial zone (EQ) at Lati-0, and the south-equatorial zone (SEQ) at longitude 80° E.

We collected Radiolarian specimens from the plankton net tow samples from 44 sampling stations in the eastern Indian Ocean (80.00°–96.10° E, 10.08° N–6.00° S). The study area covers the three transect zones between 10.08° N and 6.00° S—namely, the north equatorial transect at 90° E, the south equatorial transect (north–south lines) at 80° E, and the equatorial transect at Lat-0 (Figure 1). All the specimens were collected using a modified Indian Ocean standard plankton net (20 µm mesh size, 0.57 m diameter, 470 cm long, with a mechanical flow meter) by towing the vertical haul at a 200 m depth to the surface. The samples were preserved with 2% formaldehyde and kept at room temperature for 24 h at Dr. Sun’s laboratory (Tianjin University of Marine Science and Technology, China) for further analysis.

### 2.2. Sample Processing and Microscopic Analysis

Observation and photography were performed at 200× and 400× magnification by using an inverted optical microscope (Motic, AE 2000, Xiamen, China) which was equipped with a digital camera (Moticam 2506 (5.0 m, pixel), Xiamen, China). SEM micrographs were obtained using scanning electron microscopy (SEM). Under SEM observation, 5 mL of secondary samples were filtered on polycarbonate filter paper (0.6 m), air-dried on a stub sputtering machine, then examined by the Jeol-JSM-IT300 SEM (Jeol Ltd., Tokyo, Japan). We used target and automatic analysis software SEMs using motion light microscopy (IT300 JSM Version 1:170; Tokyo, Japan). Morphological identification and description were performed according to the traditional Radiolarian references [12,17,18,19] and classification was performed by Adl [9].

## 3. Results and Discussion

### 3.1. Radiolarian Protist in the Eastern Indian Ocean

A total of 168 Radiolarian taxa belonging to the phyla Retaria and Cercozoa were identified in the eastern Indian Ocean. The Radiolaria were comprised of 3 classes, 5 orders, 3 subclasses, 17 families, 46 genera, and 52 species; the Pheodarians were classified into 2 classes/subclasses, 1 order, 7 families, 10 genera, and 10 species (Table 1). In this study, we recorded about 60 newly reported species from the groups (Acanthria, Collodaria Taxopodida, Polycystinea, and Pheodarian). Here, we described the morphotypes, classifications, keynotes, origins, and new geographical records of species recorded in the eastern Indian Ocean.

### 3.2. Systematics and Morphology of Acantharia in the Eastern Indian Ocean

Acanthria are the celestine-bearing shell organisms; they are mostly planktonic, as described by Mueller in 1856 or 1858 [20,21]. Haeckel in 1887 initially reported 372 species of Acantharea in the phylum Rhizaria [6]; since 1887, the list of Acantharian species has been revised several times and new genera have been introduced as well as many species has been removed from the group [22,23,24,25]. Recently, only 150 species from 50 families have been validated [20,26].

**Figure 2:**
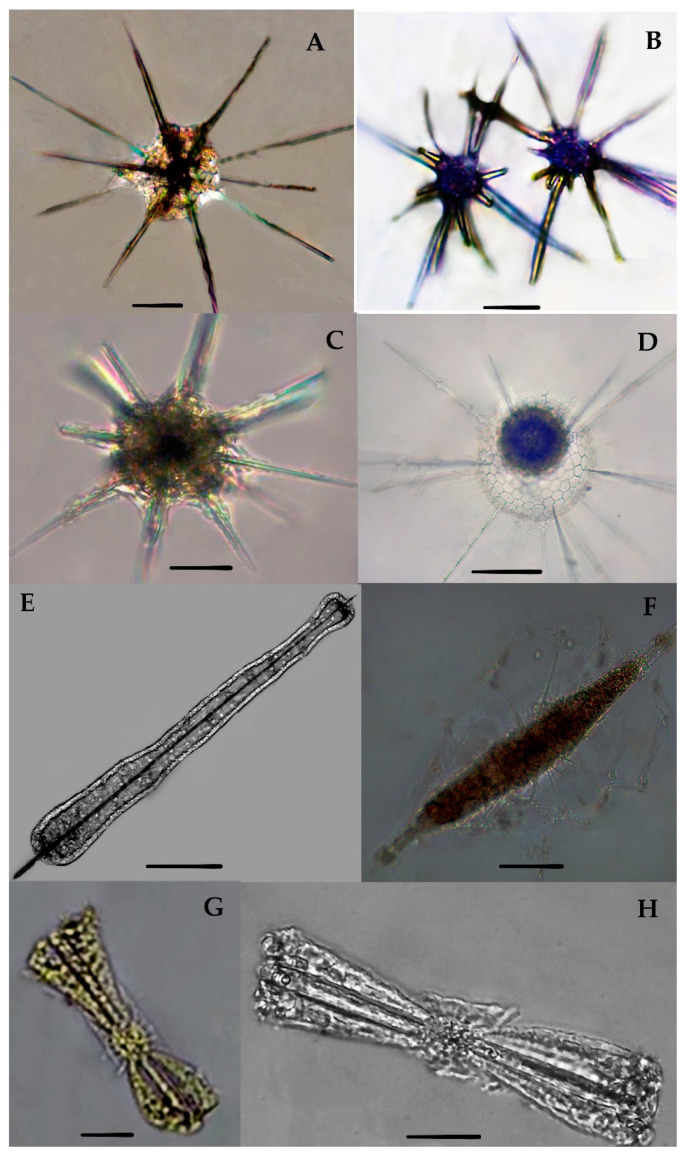
Photomicrographs of Acantharian species (**A**–**G**). The images have been modified from Munir et al. [27]. (**A**) *Acanthochiasma fusiforme*, (**B**) *Trizona brandti* and (**C**) *Acanthostaurus conacanthus* have asteroid-shaped shells with a central capsule and 10–20 radical spines.(**D**) *Dictyacantha tabulate/Tessaropelmida* has a spherical shell with mesh-type central capsules and thin spines, (**E**) *Amphilonchea elongate/Amphilithium clavarium/Amphilithium concretum* has an elongated shell with 2 apical spines and shorts spines in the central capsular shell,(**E**) is the broken cells; contains 2 apical spines, (**F**) *Amphibelone* cf. *Anomala,* (**G**) *Diploconnus faces*, (**H**) *Diploconnus cylindericus* have a bell-shaped shell with a short capsular area containing short radical spines and prominent long cornets. Scale bars: 10–50 mm.

Acanthreans consist of a star-shaped shell which is the mineral skeleton, particularly made up of strontium sulfate. The characteristic features are based on (1) the presence of a “central capsule” containing 10–20 radical spicules or spines (2), a pigmented or transparent cytoplasmic area, and (3) the presence of Apopyses in most species [21]. Based on these characteristics, about seven Acantharean species have been described in the eastern Indian Ocean (Figure 2). The morphological characteristics and size measurements of seven Acantharean species are summarized in Table 2.

### 3.3. Types of Acantharian Species and Distribution in the Eastern Indian Ocean

**Figure 3:**
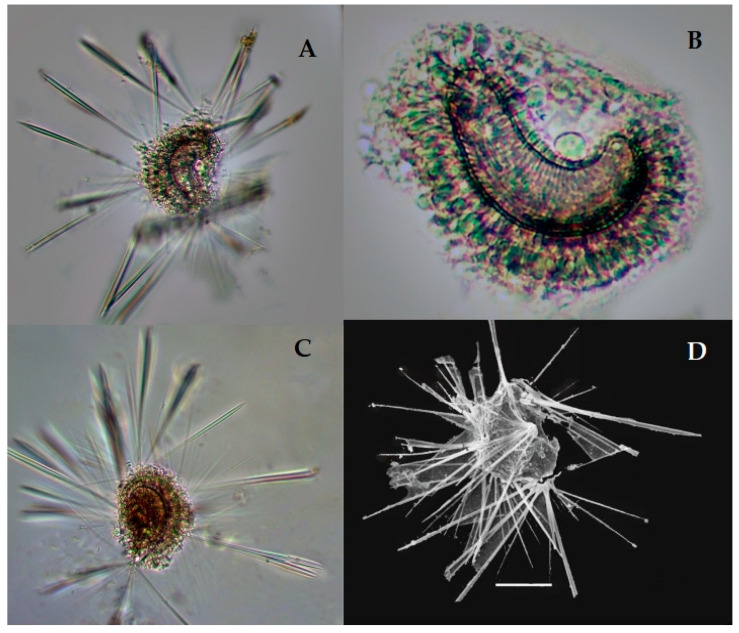
Photomicrographs of *Stichonchlea zanchlea*-Taxopodida (**A**–**D**). The images were modified from the Munir et al. [27] (**A**,**C**,**D**) complete cell showing the nuclear-with Oar-thick spines. (**B**) Cell showing nucleus with the axonemes at the central point. (**D**) SEM of thick, Oar-like axopodia spines. Scale bars: 10 µm−50 µm.

**Figure 4:**
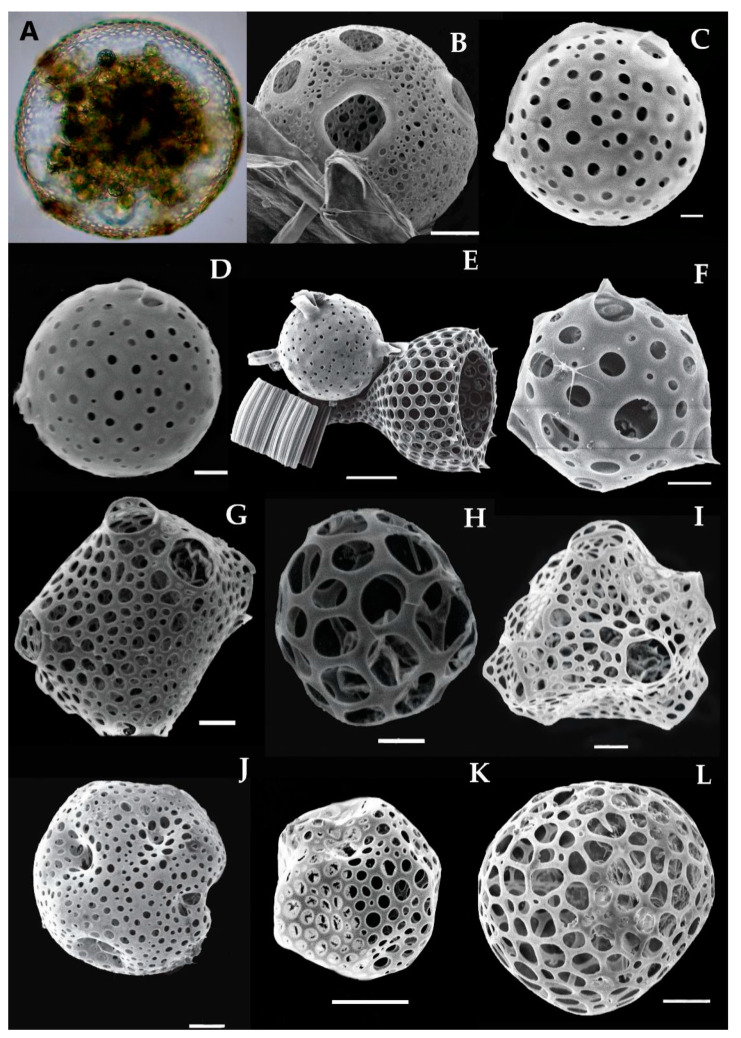
Light and scanning electron micrographs of collosphaerea and siphonsphaera species in the eastern Indian Ocean. Images were reused from Munir et al. [27]. (**A**,**B**) *Siphonosphaera magnisphaera* has large pores shell. (**C**,**D**) *Siphonosphaera polysiphonia* (**E**) *Siphonosphaera socialis,* (**F**–**J**) *Collosphaera macropora,* (**I**) *Disolenia zanguebarica,* (**K**) *Collosphaera tuberosa,* and (**L**) *Collosphaera huxleyi.* Scale bars 10, 20 and 50 μm.

About seven Acantharean species were collected from the study area. The identified species can be described as having a star-shaped shell with more than 10 or 20 needle-shape axopodial spines (Figure 2A–C); (2), a spherical shell with a polygonal mesh surface and thin spines (Figure 2D), (3) an elongated shell with two axopodia spines (Figure 2E,F), and (4) a bell-shaped shell with a cornet and ribs (Figure 2G,H).

Two species—e.g., *Acanthostaurus conacanthus* and *Acanthochiasma fusiforme*—that are very similar in morphology (Figure 2A,C) can be differentiated by their adjacent 10 diametrical spines (Table 2). The spines are shorter in *Acanthostaurus conacanthus,* which helps to distinguish them from *Acanthochiasma fusiforme* (Figure 2A–C). The *T. brandti* shell appears in pair shells, which have a central capsule and contain at least 20 radical spines (Figure 2B). Three species—e.g., *A. anomala*, *A. clavarium,* and *A.* cf. *concretum*—have an elliptical and short capsular shell with conical spines and long fan-like cornets at both apical axes (Figure 2E,F). The defining characteristics of *A. clavarium/A. concretum* are two large apical spines and a central area, which contains numerous nuclei inside the intracasular shell. *D. faces* and *D. cylindricus* are members of Diplocoonidae family and can be defined by their bell-shaped shell; both species have an elliptical shell with a minute intercapsular area which contains numerous spines which are aligned with long cornets (Figure 2G–H). The long cornet and short intracapsular shell are the main features used to differentiate the *D. faces* and *D. cylindricus* species (Table 2).

#### 3.3.1. *Acanthochiasma fusiforme* Haeckel 1861 (Figure 2A)

*Acanthochiasma fusiforme* was described by Haeckel in 1877 in the surface sediments of the Mediterranean Sea, the Atlantic, and the Pacific Ocean [25]. This species has an asteroid shell with 10 or more diametrical spines. The single shell is about 0.035 mm in size and characteristically has two cytoplasmic portions—i.e., an intercapsulum and extracapsular shell. The extracapsular shell is a yellow pale color, while the intracapsular shell is mostly transparent. From the capsular shell, the axopodia spines arise; they are slightly fusiform as well as needle-shaped and are 0.57 mm long (Figure 2A). The length of the spine and the breadth of the capsular shell are the same as those of originally reported for the species during the challenger expenditure report of Haeckel in 1877. This species was found for the first time in the eastern Indian Ocean and collected at one station—namely, St. I609—at the longitude of 90° E (Figure 1).

#### 3.3.2. *Trizona brandti* Popofsky 1904 (Figure 2B)

Our specimen of *Trizona brandti* has a star-shaped shell and can be identified by its fixed needle-shaped spines, which are almost 10 in number and cross the cytoplasm shell. The shell is surrounded by cement segments and the yellowish extra-capsulum wall (Figure 2B). The characteristics of this species also closely resemble those of the Atlantic species described by [25]. Originally, the species was described by Popofsky in 1904 in the Mediterranean Sea, and later these species were reported in several locations, including the Mediterranean Sea, the Red Sea, the English Channel, the Atlantic Ocean, and the west Pacific Ocean [25]. This species was sighted for the first time at one station (St. I609) at a longitude of 90° E in the eastern Indian Ocean (Figure 1).

#### 3.3.3. *Acanthostaurus conacanthus* Haeckel 1887 (Figure 2C)

*A. conacanthus* can be defined by a single shell which has four conical and cylindrical equatorial spines tapering from a thick base to a simple apex, as well as a large leaf-cross (Figure 2C). The total shell diameter is 0.16–0.25 mm and the spines are 0.18–0.14 mm long. This species was described by Haeckel in 1887 and isolated from the surface sediments of the south Atlantic Ocean [6]. This species has a restricted distribution area, such as the Atlantic Ocean [25]. This species was observed for the first time in the eastern Indian Ocean and collected from stations (I609, I807) at a longitude 90° E (Figure 1).

#### 3.3.4. Dictyacantha tabulate/Tessaropelmida Haeckel 1886 (Figure 2D)

*Dictyacantha Tabulate* or *Tessaropelmida* has a spherical shell that can be identified by the presence of polygonal meshes around the shell and rectangular pores. The shell has long and thin four-edged bars (0.18 mm). The intracapsular shell is a dark brown shade enclosed by a thick capsular wall (Figure 2D). The total shell length is 0.26 mm and the bars are 0.18 mm long. This species was first described by Haeckel in 1886 in the Pacific Ocean [6]. This species occurred at two stations—namely I607 and I609—in the eastern Indian Ocean (Figure 1).

#### 3.3.5. Amphilonche elongata Muller, 1858/Amphilonche cf. concretum/Amphilithium cf. clavarium Haeckel 1887 (Figure 2E)

*Amphilonche elongata/Amphilonche* cf. *concretum/Amphilithium* cf. *clavarium*/belong to the family Amphilithidae, in which the organisms mostly have cylindrical, elongated, and compressed-shape shells. The species have conical spines at the distal end of the central capsule. Specifically, two apical spines which arise from the central capsular shell are longer (Figure 2E). Numerous nuclei can be seen inside the intercapsular shell. This species resemblance to *Amphilonche elongata* and could be belongs to the *Amphilonche* cf. *concretum/Amphilithium* cf. *clavarium* Haeckel 1887. Haeckel in 1887 reported the Amphilonche species with eight short spines which were scarcely about 1/4th long, these spines may be broken in the collected specimen of the eastern Indian Ocean (Figure 2E). Originally, this species was described by Haeckel in 1887 in the Mediterranean Sea, the Atlantic, and the Pacific Ocean [6]. This species occurred at the stations I507 and I509 in the eastern Indian Ocean (Figure 1). Our specimens are the first reported from the eastern Indian Ocean.

#### 3.3.6. *Amphibelone* cf. *anomala* Haeckel 1887 (Figure 2F)

The shell consists of two equatorial spines which are small in size and the same length. The anterior ones with four spicules and the posterior ones have three thick, wing-like extensions, and spicules emerge from the central capsule. This species occurred for the first time at the stations I809 and I509 in the eastern Indian Ocean (Figure 1). This species is well known in the Australian waters [6].

#### 3.3.7. *Diploconus faces* Haeckel 1862 (Figure 2G)

Our observation was based on a bell-shaped individual which contained cornets and short pin spines (Figure 2G), which is close to the original description by Boltovskoy [26]. This species was described by Haeckel in material collected from the north Pacific Ocean [6]. This species was seen at two stations—namely I308 and I505—for the first time in the eastern Indian Ocean (Figure 1). Previously, this species was known to be widely distributed in the north Pacific Ocean, south Pacific Ocean, Atlantic Ocean, and Indian Ocean [6].

#### 3.3.8. *Diploconus cylindrus* Haeckel 1887 (Figure 2H)

This species belongs to the family Diploconidae and has an elliptical, compressed-shaped shell defined by an elongated cornet with a short intercapsulum shell that has numerous spines covered by a capsular wall. Two large, cylindrical/conical-shaped cornets (cor) enclosing the 3/4th of the spicules (Figure 2H). This species was described by Haeckel in 1887 in the north Atlantic Ocean [6]. Boltovoskoy [26] described this species in the Pacific Ocean and also in the Mediterranean Sea. This species was seen at the stations I103, I406, I505, and I807 for the first time in the eastern Indian Ocean (Figure 1).

### 3.4. Systematics and Morphology of Taxopodida Species and Distribution in the Eastern Indian Ocean

Taxopodia are organisms with oar-like movable axopodial spines and are devoid of the skeletal shell. The first specimen of Taxopodian plankton was described by Stainsy in 1909 in the Atlantic Ocean [28]. It was described as *Sticholonche ventricosa* by Meunier in 1910 [29]. Hertwig 1887 [30] introduced the first family, Sticholonchida, which has been revised by other scientists since 1908 [8,31,32,33]. In the beginning, they were placed in the Helizoa group, which included the Polycystine and Pheodaria groups; later, the characteristics of Taxopodia were found to be distinctly different and they were separated to the new order, Taxopodida [8,34]; only one type of species, *Sticholonche zanchlea*, has been validated in the Taxopodian group [11].

### 3.5. Type of Taxopodida Species and Distribution in the Eastern Indian Ocean

#### *Sticholonche zanclea* Hertwig 1887 (Figure 3A–D)

Syn: *Sticholonche ventricosa* Meunier, 1910, Figure 3, pl. 22.

*Sticholonche zanclea* is a plankton species that has a non-skeletons shell and an oar-like structure which contains thick axopodia spines (Figure 3A–D). This species was recently found in the eastern Indian Ocean at the stations I101, I103, I105, I107, I109, I302, I404, I407, I412, I507, I509, I611, I809, I811, I815, and HF01; see Figure 1. This species has abundantly been reported from the East China Sea [8,35]. However, this is the first record of *Sticholonche zanchlea* in the eastern Indian Ocean.

### 3.6. Systematics and Morphology of Polycystinea Radiolarian in the Eastern Indian Ocean

**Figure 5:**
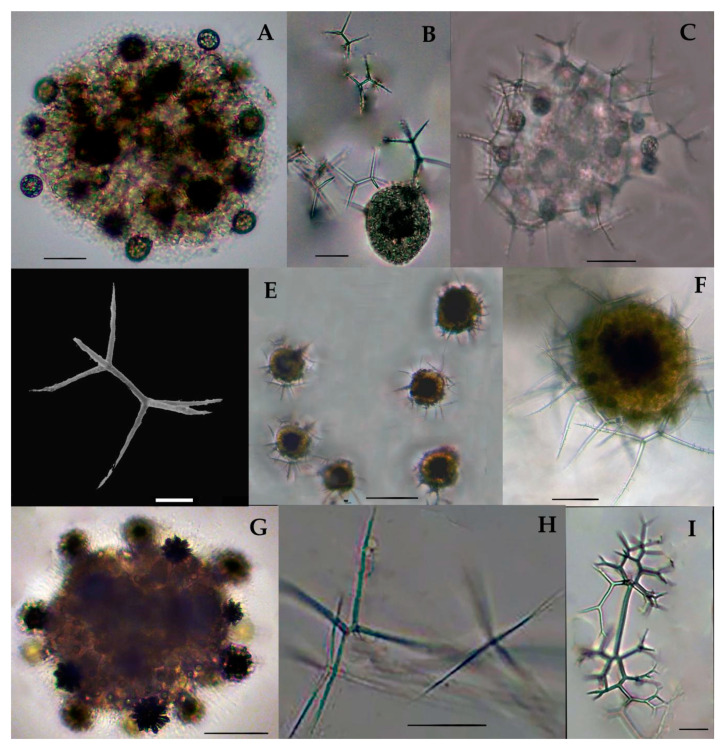
Photomicrographs of Collozoum (**A**) and Sphaerozoum (**B**–**G**) and Thallosoxanthium species (**H**,**I**). The images were modified from the Munir et al. [27]. (**A**) *Collozoum inerme* show non-spicule cells; (**B**–**D***) Sphaerozum punctatum* show colonies and have intercapsulums with spicules; (**E**,**F**) *Sphaerozum fuscum* is formed of colonies of spherical shells with spicules; (**G**) *Sphaerozum ovodimare* has four ray spicules; (**H**) *Thalassoxanthium cervicorne* has two shanks at the end of the middle rod; (**I**) *Thalassoxanthium octoceras* has a four-triangular shank at both ends of the intermediate rod. Scale bars are 10 μm and 50 μm.

**Figure 6:**
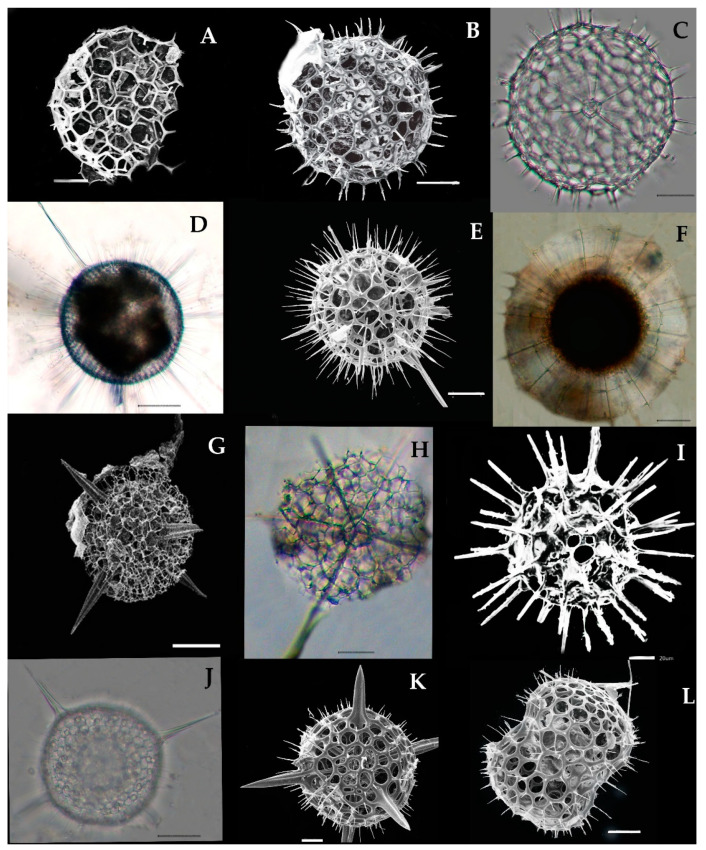
Light and scanning electron micrographs of Spumellarian species (**A**–**L**). The images were modified from Munir et al. [27]. (**A**) *Acanthosphaera actinota*; (**B**) *Acanthosphaera pinchuda*; (**C**,**D**) *Actinosphaera capillacea,* (**E**) *Actinosphaera tenella*, (**F**) *Arachnosphaera myriacantha*, (**G**) *Cromyechinus circumtextum*, (**H**) *Centrocubus cladostylus,* (**I**) *Elatomma penicillus,* (**J**,**K**) *Hexalonche amphisiphon,* (**L**) *Didymocyrtis tetrathalamus.* Scale bars: 10 μm, 20 μm, 100 μm.

**Figure 7:**
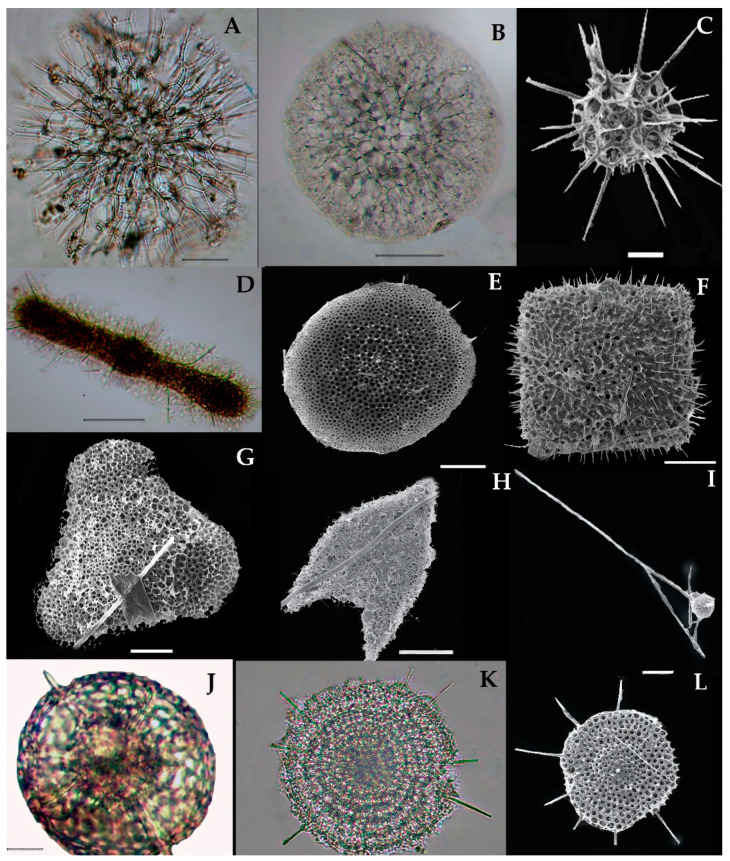
Photomicrographs of Spumellarian species in the eastern Indian Ocean. Images were modified from Munir et al. [27]. (**A**) *Styptosphaera spongiacea*, (**B**) *Spongodictyon spongiosum*, (**C**) *Streblacantha circumtexta, (***D***) Spongurus cylindricus*, (**E**) *Spongurus pylomaticus,* (**F**) *Spongaster tetras tetras, (***G***) Spongodiscus sp.,* (**H**) *Euchitonia elegans,* (**I**) *Xiphosphaera tessaractis,* (**J**) *Xiphatractus sp.*, (**K**) *Stylodictya multispina,* (**L**) *Stylochlamydium venustum.* Scale bars: 10 μm, 20 μm, 100 μm.

**Figure 8:**
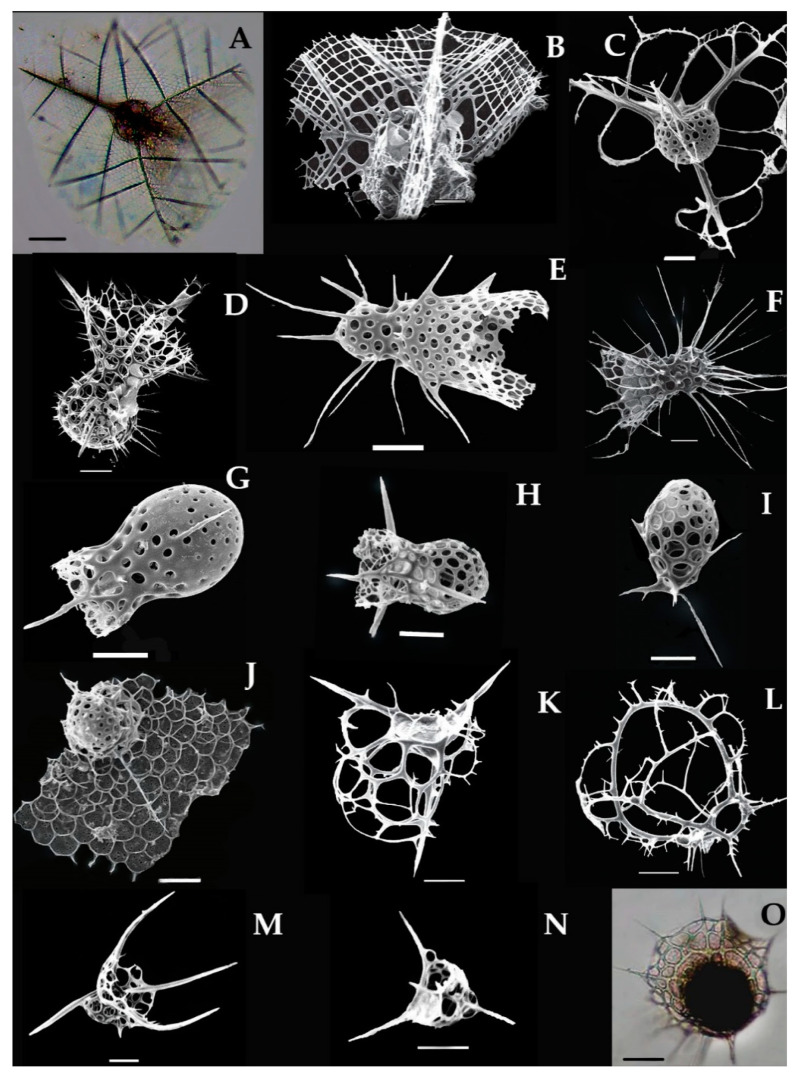
Light and scanning electron micrographs of Nassellarian species (**A**–**O**). These images were modified from Munir et al. [27]. (**A**,**B**) *Callimitra emmae* (**C**) *Clathrocorys murrayi*, (**D,H**) *Lophophaena capito*, (**E**,**F**) *Lophophaena cylindrica*, (**G**) *Peromelissa phalacra,* (**I**) *Peridium spinipes*, (**J**) *Lampromitra schultzei,* (**k**) *Phormacantha hystrix*, (**L**) *Plectacantha trichoides*, (**M**) *Pseudodictyophimus gracilipes*, (**N**) *Cladoscenium ancoratum*, (**O**) *Tetraphormis dodecaster.* Scale bars: LM: 10 μm and SEM: 10 μm, 20 μm.

**Figure 9:**
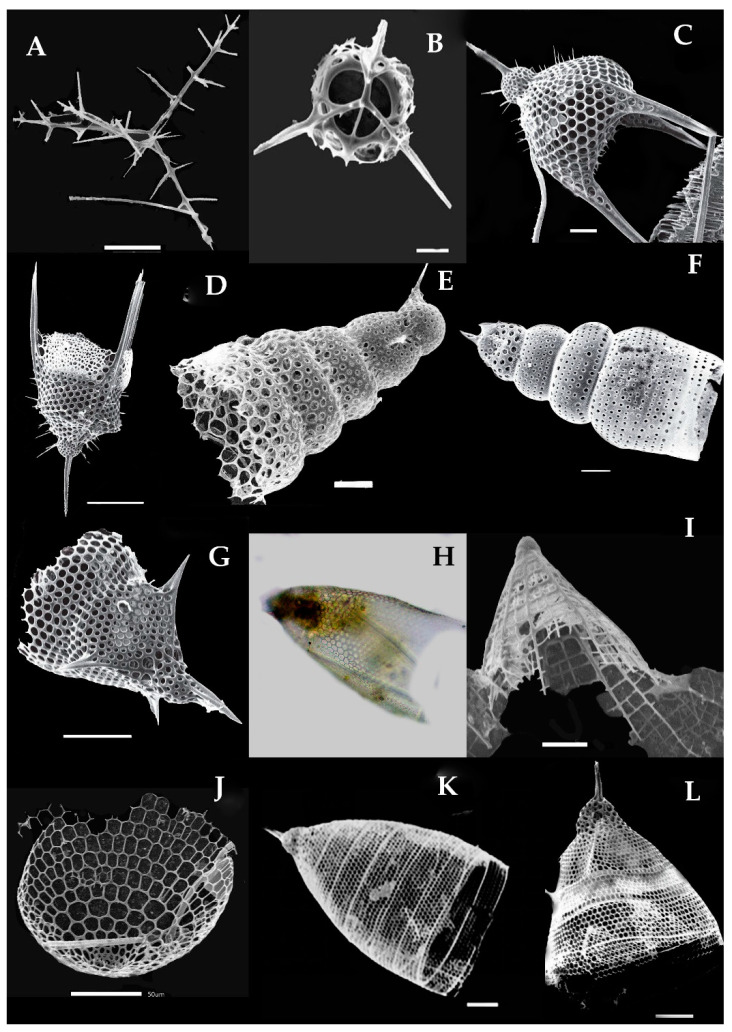
Light and scanning electron micrographs of Nassellarian species (**A**–**L**). The images were modified from Munir et al. [27]. (**A**) *Tetraplecta pinigera*; (**B**) *Archibursa tripodiscus;* (**C**) *Pterocanium praetextum,* (**D**) *Pterocanium korotnevi;* (**E**) *Triacartus undulatum*, (**F**) *Spirocyrtis subscalaris*, (**G**) *Pterocorys hertwigii*; (**H**) *Conarachnium parabolicum;* (**I**) *Litharachnium tentorium;* (**J**) *Eucecryphalus clinatus;* (**K**) *Eucyrtidium dictyopodium;* (**L**) *Dictyocodon palladius;* Scale bar: 10 µm, 20 µm and 50 µm.

**Figure 10:**
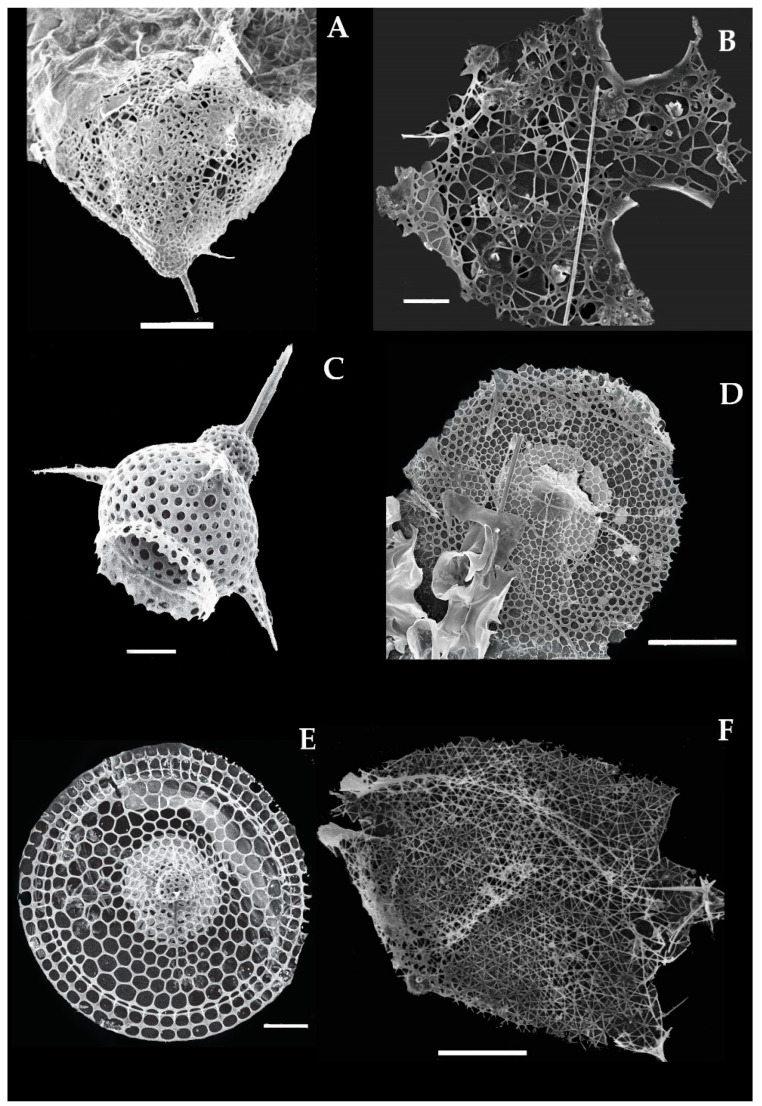
Light and scanning electron micrographs of Nassellarian species (**A**–**F**) These images were modified from Munir et al. [27]. (**A**,**B**) *Sethoconus venosus,* (**C**) *Theocorys veneris,* (**D**) *Theophormis callipilium*, (**E**) *Theopilium tricostatum*; (**F**) *Cephalospyris cancellate.* Scale bar: 10 µm, 20 µm, 50 µm.

More than 400 species of the polycystina group have been identified globally throughout the World Ocean [36]. Recently, 16 species of Collodarians (Figure 4 and Figure 5), 18 species of Spumellarians (Figure 6 and Figure 7), and 75 species of Nassellarians (Figure 8, Figure 9 and Figure 10) were collected from the eastern Indian Ocean. Morphologically, a symmetrical or concentric shell either in single or pair form has appeared, along with numerous radial or needle-shaped spines—e.g., Spumellaria species (Figure 6 and Figure 7). A species with asymmetrical, concentric shells can be described by having a well-developed cephalis, thorax, and abdomen. These portions are interconnected by the median arch, and there are apical and ventral or lateral spines with medial bars—e.g., the Nasellarian species (Figure 8, Figure 9 and Figure 10). Nassellarians have specific apophyses on their lateral spines, while their cervical and pectoral area have an antecephalic lobe, eucephalic lobe, postcephalic lobe, and lateral lobe. There are some non-skeleton shell species which have spicules embedded in their gelatinous matrix—e.g., the Collodaria species (Figure 4 and Figure 5).

### 3.7. Types of Collodarian Species and Distribution in the Eastern Indian Ocean

Collodaria are mostly found in the form of colonies and also in solitary cells. These are heterotrophic marine protists found in the oligotrophic region [37]. Generally, collodarians lack axopodial spines (Haeckel, 1887). The cells contain spicules around the central capsule and are included in the families Sphaerozoidae, Siphonosphaeridae, and Collosphaeridae. Species of these families are found in abundance in the upper water column [37]. In the eastern Indian Ocean, we found a variety of *Siphosphera* species—e.g., *Siphonosphaera polysiphonia*, *Siphonosphaera socialis*, *Solenosphaera zanguebarica*, *Collosphaera macropora,* and *Collosphaera tuberosa* (Table 1; Figure 4). All of them are Collosphaeridae and only the first two are Siphonosphaera. Most of these have been previously reported in the same region [16]. Only *Siphonosphaera magnisphera* Takahashi was recorded for the first time in the eastern Indian Ocean. This species of the genus *Siphonosphaera* has the base of the spherical shell and lacks the axopodial spines. However, there are large pores on the surface of *S. magnisphera* that can differentiate this from other species (Figure 4A,B). Species of Sphaerozoidea include *Sphaerozoum punctatum, Sphaerozoum fuscum,* and *Sphaerozoum ovodimare*, which occur in the eastern Indian Ocean. Sphaerozoum shells are spicular and not spherical (Figure 5). The cells of *S. punctatum* contain rod-shaped spines (Figure 5C,D) which have a close resemblance to other Sphaerozoum species. Only short-rumbled spines with pointed needles at the end may discern these species. *Sphaerozoum fuscum* has broad pin-shaped spicules around a gelatinous matrix shell (Figure 5F), and the three ray spicules in the *S. punctatum* species can distinguish it from the *Sphaerozoum ovodimare* species (Figure 5G) which has four different sizes of ray spicules (Table 2). Thalassoxanthihum species such as *Thalassoxanthium cervicorne* and *Thalassoxanthium octoceras* were rarely found in a recent study of the eastern Indian Ocean (Figure 5H,I). *Thalassoxanthium cervicorne* has been reported in the central Pacific Ocean, while *Thalassoxanthium octoceras* has been reported in Madagascar and the Indian Ocean [6].

#### 3.7.1. *Siphonosphaera magnisphaera* Takahashi 1991 (Figure 4A,B)

Takahashi described the *S. magnisphaera* from the Atlantic Ocean [17]. Its spherical shell contains numerous circular large pores and small pores (Figure 4A,B). The macropores are large as ¼–1/2 in length than the whole shell diameter [17]. This species has not been observed with spicules or spines (Figure 4C–O). This species was reported for the first time at one station (I609) in the eastern Indian Ocean (Figure 1).

#### 3.7.2. *Collozoum inerme* J. Müller 1862 (Figure 5A)

Syn. *Sphaerozoum inerme* J. Müller, 1856, p. 478.

*Collozoum inerme* was described by Muller in 1856 [20] as *Sphaerozoum inerme.* Characteristically, the species has no proper spicules or skeleton shell, and the cell contains numerous round cell boundaries around the gelatinous matrix (Figure 5A). The total cell length is 0.239 mm. This is a tropical Pacific species that has a wide distribution from the northern Norwegian Sea [38] to the south Atlantic Ocean [25,37]. This species was recorded for the first time at the stations I815, I103, I107, and I609 in the eastern Indian Ocean (Figure 1).

#### 3.7.3. *Sphaerozoum punctatum* (Huxley) Müeller 1858 (Figure 5B–D)

Syn.*Sphaerozoum geminatum* Haeckel 1887, p.45, Figure 4.
*Thalassicola punctata* Huxley 1851, p. 434; pl. 16, Figures 1–3.
*Plagonium* cf. *Sphaerozoum* Benson, 1966, p. 286–287; pl. 19, Figures 12 and 13.

This species was described by Haeckel in 1887 in the Mediterranean Sea and the Central Pacific Ocean [39]. *S. punctatum* are colonies that form shells that contain rod-shaped spicules which are embedded in the gelatinous matrix (Figure 5D). Each spicule has three ray structure spines that emerge from both ends. The total shell length is 0.22 mm and the spicules are 0.096 mm long. Huxley 1851 [40] named it *Thalassicola punctata* and Benson named this species *Plagonium* cf. *Sphaerozoum* in 1966 [41]. Haeckel 1887 described the *Sphaerozoum germinatum* as another species, which is now named as *S. punctatum* according to Müeller [11,41,42]. In the eastern Indian Ocean, *S. punctatum* is frequently recorded at stations (Stas. I09, I308, I310, I314, I318, I403, I412, I413; I501, I503, I509, I511, I609, HF05) (Figure 1). This species has been reported earlier in the southern Indian Ocean [6,43].

#### 3.7.4. *Sphaerozoum fuscum* Meyen 1834 (Figure 5E,F)

This species has been described before by Meyen in 1934 in the Pacific Ocean [44]. This species appeared as the colonies containing the spicules which were embedded in the gelatin matrix (Figure 5E). The single cell has a capsular wall (CW) which contains the central vacuoles. Numerous symbiotic parasites (colored) were found inside the intercapsulum area (Figure 5F). There are rod-shaped spicules around the cells which are spiny or needle-shaped. These spicules are longer compared to those of other *Sphaerozoum* species (Figure 4F). The dimensions of the cell are 0.233 mm; spicules with rods are 0.03 mm, needles are 0.14 mm, small lateral branches are 0.0036 mm, and symbiont parasites are 0.026 mm. In the eastern Indian Ocean, this species was recorded for the first time at the one station (I312) in the eastern Indian Ocean (Figure 1); previously, this species was reported in the East China Sea.

#### 3.7.5. *Sphaerozoum ovodimare* Haeckel 1860 (Figure 5G)

Haeckel in 1860 described this species in the Mediterranean Sea, Naples, Messina, the Atlantic Ocean, the Canary Islands, the Cape Verde Islands, and off the west coast of Africa [6,39]. This species has four-rayed spicules that are like thorns which are loosely attached to cellaeform bodies inside the capsular wall (Figure 5G); it also contains symbiont parasites inside the intracapsular area. The size of each spicule is 0.0315 mm. This species bears a close resemblance to *S. punctatum* and has been known since 1862. Only the long shank in *S. punctatum* differentiates it from *S. ovodimare* (Figure 5G). In the eastern Indian Ocean, this species was recorded for the first time at the stations I611, I609, and I603 (Figure 1).

#### 3.7.6. *Thalassoxanthium cervicorne* Haeckel 1862 (Figure 5H)

The *Thalassoxanthium cervicorne* was described by Haeckel in 1862 in the central Pacific Ocean [39]. This species can be identified on the basis of its spicules, which are divided into three tri-angle shanks of the same size. The three triangles branch out at the common point of the distal end. These branches are forked once or twice (Figure 5H). The thin, unequal, and bent-shaped bifurcation is almost the corner horn. The size of the spicules with the triangle shanks is 0.234 mm and branches are 0.0086–0.00152 mm. This species was seen at the station I607 for the first time in the eastern Indian Ocean (Figure 1).

#### 3.7.7. *Thalassoxanthium octoceras* Haeckel 1887 (Figure 5I)

The *Thalassoxanthium octoceras* was described by Haeckel in 1887 in Madagascar and Rabbe Island in the Indian Ocean [6]. This species can be identified on the basis of its spicula, which have a short/intermediate rod with four diverging shanks arising at each end (Figure 5I). The shank is smooth and curved or bent to 4 to 8 times the length of the intermediate rod (Figure 4I). The dimension of the short intermediate rod is 0.0075 mm and the four diverging shanks are 0.109–0.1199 mm. This species occurred for the first time at the location I310 in the eastern Indian Ocean (Figure 1).

### 3.8. Systematics and Morphology of Spumellarian in the Eastern Indian Ocean

Spumellaria was identified by Ehrenberg in 1875 [45] and described on the basis of having a lattice, concentric shell, typically spherical in shape, distinguished by spicules and round pores in the capsular wall [8]. Haeckel in 1887 [6] and Riedel in 1967 [46] amended the species identification. There were 4 families and 11 species described recently in the eastern Indian Ocean.

### 3.9. Type Species of Spumellaria and Distribution in the Eastern Indian Ocean

#### 3.9.1. *Acanthosphaera actinota* Haeckel 1860 (Figure 6A)

Syn. *Heliosphaera actinota* Haeckel 1862, p. 352, pl. 9, Figure 3.
*Acanthosphaera tunis* Haeckel 1887, p. 210.
*Acanthosphaera tenuissima* (Haeckel) Renz, 1976, p. 99, pl. 2, Figure 11.
*Acanthosphaera corloca* Boltovskoy and Riedel 1980, p. 107, Figure 2 and pl. 1.

This species was originally described by Haeckel in 1860 in the south of the Indian Ocean, the central Pacific Ocean, and the Atlantic Ocean [6,39]. Initially, this species was described as *Heliosphaera actinota* and then changed into a new genus, Acanthosphera, which is valid to date [6]. This species has a small, somewhat latticed shell that contains thin meshes on the cortical shell (Figure 6A). There are large polygonal pores with thick bars (6–8) can be seen on each network node. In the eastern Indian Ocean, this species occurs frequently at the stations I101, I103, I302, I308, I312, I507, I605, I611, and I815 (Figure 1). This is the first record of *A. actinota* in the eastern Indian Ocean.

#### 3.9.2. *Acanthosphaera pinchuda* Boltovskoy, 1980 (Figure 6B)

This species was described earlier by Haeckel in 1887 and Boltovskoy in 1980 in the North Atlantic Ocean [47,48,49]. This species can be characterized by its thin cortical shell with sharp spines (Figure 6B). This species has been reported before from the Pacific and Atlantic oceans [39,49,50] and it was found for the first time at the stations I109 and I302 in the eastern Indian Ocean (Figure 1).

#### 3.9.3. *Actinomma capillaceum* Haeckel 1887 (Figure 6C,D)

Syn: *Actinomma sp. aff. A. arcadophorum* (Haeckel) in Takahashi and Honjo 1981, p. 147, pI. 2, Figure 4.

This species was described by Haeckel in 1887 in the central Pacific Ocean and the Atlantic Ocean [48]. This species can be observed on the basis of its cortical shell containing three-bladed spines (Figure 6C,D). The shell has polygonal grids around the short narrow wall and a triangular pyramid from the medullary shell. The pores in the medullary shell are 0.0149 mm smaller than those in the cortex shell. The three-bladed spines are 0.024 mm in size. This species was found at the stations I301, I401, and I809 for the first time in the eastern Indian Ocean (Figure 1).

#### 3.9.4. *Actinosphaera tenella* (Haeckel) Hollande and Enjumet, 1960 (Figure 6E)

Syn: *Haliomma tenellum* Haeckel, 1862, p. 428; 1887, p. 236.
*Haliomma spinulosa aff* in Müller, 1858a, p. 40, pi. 4, Figure 7.
*Actinosphaera capillaceum* (Haeckel) in Hollande and Enjumet, 1960, pi. 52, Figure 3.

This species was described as *Haliomma tenellum* by Haeckel in 1862 in the South Pacific Ocean [38]. This species is characterized by a concentric shell with a single radial bar that contains a number of bristle spines (Figure 6E). The cortix shell is comparatively larger than a medullary shell and has irregular pores with thin bars. The bristle-shaped spines are somewhat straight. The measurements of the radial spine are 0.12 mm and bristle spines are 0.1002 mm. This species can be confused with a species with a close resemblance, *A. capillaceum*, due to the thin bars that are situated at the cortix shell. Previously, this species was reported in the Mediterranean Sea and the douth Atlantic Ocean [17,39]; it occurred for the first time at the one station (St. I101) in the eastern Indian Ocean (Figure 1).

#### 3.9.5. *Arachnosphaera myriacantha* Haeckel 1860 (Figure 6F)

Syn: *Arachnosphaera hexasphaera* Popofsky 1912, p. 108, Figures 19–21;
*Arachnosphaera hexasphaera* Takahashi and Honjo 1981, p. 147, pI. 2, Figure 13.

*Arachnosphaera myriacantha* was described by Haeckel in 1860 from the equatorial region of the Pacific Ocean [47]. This species has a concentric shell that is spherical in shape with a cobweb network of concentric spines. The hexagonal meshes and cylindrical spines arise from its nodes. This species was recently recorded for the first time at the stations I308, I310, I312, I314, I318, I412, I413, I501, I507, and I511 in the eastern Indian Ocean (Figure 1).

#### 3.9.6. *Cromyomma circumtextum* Haeckel 1887 (Figure 6G)

*Cromyomma circumtextum* was described by Haeckel in 1887 in the South Atlantic Ocean [6]. This species can be characterized as having a concentric shell, spherical in shape, which has three blade spines (Figure 6G). The surface contains numerous irregular spines around the polygonal meshes and also contains thin thread-like bars (Figure 6G). This species has a wide distribution in the Southern Ocean and the Pacific Ocean [26], and it was recently recorded for the first time at the stations I607 and I807 in the eastern Indian Ocean (Figure 1).

#### 3.9.7. *Centrocubus cladostylus* Haeckel 1887 (Figure 6H)

Syn: *Centrocubus octostylus* (Haeckel), Takahashi, 1991, P. 191, Pl. 7, Figure 1.
*Octodendron pinetum* (Haeckel) in Boltovskoy and Riedel, 1980, p. 113, Pl. 3, Figure 2A,B.

*Centrocubus cladostylus* was described by Haeckel in 1887 in the North Atlantic Ocean [6]. This species is characterized by a sponge-like network of cortical shells which contain about eight radial spines. These spines are club-shaped and also contain at least 24 secondary spines (Figure 6H). The measurements of the medullary shell, polygonal pores, and radial spines are 0.0030, 0.0018, and 0.012 mm, respectively. This species was previously reported in the south and north Pacific, the southwest Indian Ocean, and the south Atlantic Ocean [26,39]; it was recorded for the first time at the stations I101, I501, I509, I511, I605, and I609 in the eastern Indian Ocean (Figure 1).

#### 3.9.8. *Elatomma penicillus* Haeckel 1887 (Figure 6I)

*Elatomma penicillus* was described by Haeckel in 1887 off the west coast of Norway, Bergen [6]. The original description was based on its delicate shell, which has thick bars at the polygonal pores in the medullary shell. The shells contain thick bars including 20 or more thin beans. They extend outwards with short brush-like bundles with a radius of 20 to 9, and extend to twenty straight triangular prismatic radial spines which have irregular branches at the end (Figure 6I). This species was found at one station (St. I314) for the first time in the eastern Indian Ocean (Figure 1).

#### 3.9.9. *Hexalonche amphisiphon* Haeckel 1887 (Figure 6J,K)

Two species—e.g., *Hexacontium armatum-hostile* and *Hexalonche amphisiphon*—were recently recorded in the eastern Indian Ocean (Table 1). *H. amphisiphon* can be characterized by thin bars, six main spines, and hexagonal pores (Figure 6J,K). The measurements of the shell, hexagonal pores, and six radial spines are 0.523, 0.039, and 0.27 mm, respectively. *H. amphisiphon* was described by Haeckel in 1887 from the Central Pacific Ocean [6] and its distribution was expanded from the north Atlantic Ocean [17] to the equatorial Pacific Ocean [39]. This species was recorded for the first time at the stations I101, I308, I609, and I607 in the eastern Indian Ocean (Figure 1).

#### 3.9.10. *Styptosphæra spongiacea* Haeckel 1887 (Figure 7A)

This species was described by Haeckel in 1887 in the central Pacific Ocean [6]. The species has spongy meshes on the concentric shell at the midpoint. This is more compact than the peripheral part and becomes looser at the rough surface (Figure 7A). The measurements of the spongy network and medium pores are 0.068 and 0.046 mm, respectively. Previously, this species was reported in the south Atlantic Ocean [17] and recorded for the first time at the locations I308, I503, I607, and I809 in the eastern Indian Ocean (Figure 1).

#### 3.9.11. *Streblacantha circumtexta* Jorgensen 1910 (Figure 7C)

Syn. *Sorolarcus circumtextus* Jorgensen 1910, p. 121, pl. 11, 12 Figure 46.

This species was described by Jorgensen in the Norwegian Sea in 1900 [17]. This asymmetric shell has compact-needle shape radial spines. This oval contains numerous pores from small to large sizes (Figure 7C). This species was named *Sorolarcus circumtextus* Jorgensen in 1910 by Schroder [50]. This species has been modified into the new genera *Streblacantha* [51], which has been accepted and is valid to date [6,11]. This species is distributed from the Arctic Sea to the Nordic Sea and north Atlantic Ocean [52]. Based on these reports, this species is rare in these areas and still has not been reported in the Pacific Ocean. Recently, this species was recorded for the first time at two stations (Stas. I308; I503) in the eastern Indian Ocean (Figure 1).

#### 3.9.12. *Spongurus pylomaticus* Riedel 1958 (Figure 7F)

*Spongurus pylomaticus* was described by Riedel in 1958 from sediment samples of the Antarctic Ocean [53]. This species can be characterized by its compressed shell which has a spongy framework on the dorsal sides and few dentations [53]. The bristle shape spine at this end has teeth at the pylome. The spongy form mesh surface contains small pores (Figure 7F). This species was reported in all of the oceanic regions, such as the Pacific Ocean, the Atlantic Ocean, the Indo-Pacific Ocean, and the SW part of the Indian Ocean [46,54,55]; it was recently recorded at the stations I101, I103, I601, and I609 for the first time in the eastern Indian Ocean (Figure 1).

#### 3.9.13. *Xiphosphaera tessaractis* Dreyer 1913 (Figure 7I)

*Xiphosphaera tessaractis* was described by Dreyer in 1913 in the central Pacific Ocean [56]. This species can be characterized by its spherical shell and smooth surface, which contains equally distributed pores and three radial spines, which are extended at one axis (Figure 7I). The central shell was measured as 0.12 mm and each circular pore is 0.013 mm; the three radial spines are 0.027 mm. This species was previously reported in the central Pacific Ocean and northern Arabian Sea [17], and was recently recorded for the first time at the stations I103, I109, I303, I308, and I412 in the eastern Indian Ocean (Figure 1).

#### 3.9.14. *Xiphatractus* sp. Dreyer 1913 (Figure 7J)

*Xiphatractus* sp. is matched well to the original description by Dreyer, 1913, on the basis of two small polar spines [56]. This species can be characterized by the single cortical and double medullary shell, more elliptical with the hexagonal framework, and two small radial spines. (Figure 7J). There are cortical-medullary interconnecting rods that lie in many planes. The whole shell measured 0.19 mm with inter-capsulum of 0.16 mm and extra-capsulum of 0.046 mm. The pores on the surface are circular as well as hexagonal and were measured as 0.0279 mm. Two polar spines are 0.15 mm and short, smooth, and conical in shape (Figure 7J). It is well known in the tropical Pacific and Atlantic Ocean [17]. This species recently recorded for the first time at the one station (St. HF01) in the eastern Indian Ocean (Figure 1).

#### 3.9.15. Spongotrochus longispinus (Figure 7K)

Syn: *Stylodictya multispina* Haeckel 1860, p. 842; 1862, p. 496, pi. 29, Figure 5.

*Stylodictya longispinus* was described as S. *multispina* by Haeckel in 1860 from sediment samples of the Mediterranean, the Atlantic Ocean, the Indian Ocean, and the Pacific Ocean [41]. This species was recently revised by Lazarus et al., who placed the *S. multispina* into the category of *S. longispinus* [11]. This species can be characterized by the concentric shell or wheel shape shell, which contains numerous rings from the center to the edge of radial barb (Figure 7K). The whole shell measures 0.267 mm with circular pores (0.119 mm). The rings are shorter as 8 or 12 as radiating beams from the central chamber and outside, more than 40 rings are perforated beams that originate from the edge of the bristle of 2 to 4 rings (Figure 6K). This species recorded for the first time at the station I503 in the eastern Indian Ocean (Figure 1).

#### 3.9.16. *Stylochlamydium venustum* Bailey Haeckel, 1887 (Figure 7L)

Syn. *Perichlamydium venustu*m Bailey 1856, p. 5, pl. 1, Figures 16 and 17.
*Spongotrochus (?) venustum* Bailey, Nigrini and Moore 1979, p. S119, pl 15, Figure 3a,b.
*Spongotrochus venustum* (Morley, 1985) pl. 2, Figure 1A,B.

*Stylochlamydium venustum* was originally described by Bailey Haeckel in 1856 as *Spongotrochu venustum* in the North Pacific Ocean, Kamtschatka [57]. *S. venustum* resemblances the *Stylodictya multispina* but can be distinguished from the 20–24 radial spines around the discoidal edge. The species characterized by the concentric and disk shape shell has spongy form rings located at the central point and 20–24 radial spines (Figure 7L). The whole disk was measured as (0.387 mm) with pores (0.0041–0.0061 mm). There are numerous beams with projecting spines are (0.134 mm). This species recorded for the first time at the one station (St. I605) in the eastern Indian Ocean (Figure 1).

### 3.10. Systematics and Morphology of Nassellarian in the Eastern Indian Ocean

Nassellarian was described earlier by Ehrenberg in 1875 [45] and Haeckel in 1887 [6], who amended the characterization of the species on the base of cephalic (porous helmet shape), thorax, and tripod including a sagittal ring enclosing the central capsule [6]. There are 21 species from 4 families described here which are recently recorded in the eastern Indian Ocean.

### 3.11. Types of Nassellarian and Distribution in the Eastern Indian Ocean

#### 3.11.1. *Callimitra carolotae* Haeckel, 1887 (Figure 8A,B)

Syn: *Callimitra emmae* (Haeckel), p. 1218; pl. 63, Figures 3 and 4.
*Callimitra emmae* Takahashi and Honjo, 1981 p. 151; pl. 7, Figure 11.
*Callimitra emmae* Takahashi 1991, p. 99; pl. 26, Figure 14.

This species was described as *Callimitra emmae* by Haeckel in 1887 in the Atlantic Ocean [6]. This species characterized by the subspherical cephalic which contains a mesh network and three vertical wings in a polygonal shape (Figure 8A,B). Thorax somewhat spherical from basal end to vertical wings and sines is dented at this point (Figure 8A). The size of the Pyramidal (0.0819 mm); long spines (0.076 mm). This species also reported from the Pacific Ocean [39]. There are other Callimitra species also found in the eastern Indian Ocean such as *Clathrocorys teuscheri* and *Clathrocorys murrayi* (Figure 7C–E). This species occurred from the station (St. I508) for the first time in the eastern Indian Ocean (Figure 1).

#### 3.11.2. *Lophophaena capito* Ehrenberg 1873 (Figure 8D,H)

Syn: *Lithomelissa capito* Ehrenberg 1873, p. 240.
*Lophophaena* cf. *capito* Ehrenberg in. Takahashi 1991, p. 96, Pl. 25, Figures 6–9.

*Lophophaena capito* was described by Ehrenberg in 1873 in the Pacific Ocean [45]. This species can be identified by the well-developed bulb shape cephalic that contains numerous spines and adjacent to the cylindrical thorax. The cephalic measured as 0.120 mm which has three-bladed spines (0.075 mm). These spines are large in size, have circular pores that are arranged in vertical rows on the surface (Figure 8D,H). This species commonly found in the North Atlantic Ocean, Pacific Ocean, and SW Indian Ocean [28,58]. Recently, this species recorded for the first time at the stations (Stas. I101, I105, I109, I503, I505, I815) in the eastern Indian Ocean (Figure 1).

#### 3.11.3. *Lampromitra schultzei* (Haeckel) Takahshi 1991 (Figure 8J)

Syn: *Eucecryphalus schultzei* Haeckel, 1862, p. 309, pl. 5. Figures 16–19; 1887, p. 1216.
*Lampromitra coronata* Haeckel, 1887, p. 1214, pI. 60, Figure 7.
*Sethophormis pentalactis* Haeckel in Takahashi and Honjo 1981, p. 152, pI. 8, Figure 5.

Haeckel in 1887 described this species in the Pacific and the Atlantic Ocean [6]. This species characterized by the small cephalic with conical apical spine and large, porous shape thorax in the ragged form (Figure 8J). The peristome has more than three rows that are regularly aligned and has small, sub-rectangular pores (Figure 8J). The measurements of the dome-shaped shell are (0.039 mm); pores are (0.055 mm) and spines are (0.12 mm). Other species such as *Lampromitra cracenta*, *Lampromitra danaes*, and *Lampromitra schultzei* were recorded in the eastern Indian Ocean (Table 1). This species recorded for the first time at the stations I107, I308, I310, I603 in the eastern Indian Ocean (Figure 1).

#### 3.11.4. *Plectacantha trichoides* Jørgensen 1905 (Figure 8L)

This species has the same morphology as *Phormacantha hystrix*, and was described by Jorgensen in 1905 in the Mediterranean Sea [51]. This species can be characterized by cells without pores on their surface and has many large polygonal pores and thin frame (Figure 8L). They have two lateral arches that are delicate joints to become large whole (Figure 8L). Each arched has few radial spines that have four-crossed circular spines to form secondary arches. This species has been previously reported from the North Pacific Ocean [39]. Recently, this species recorded at the stations (Stas. I302, St. I312) in the eastern Indian Ocean (Figure 1).

#### 3.11.5. *Pseudodictyophimus gracilipes* (Bailey) Petrushevskaya 1967 (Figure 8M)

Syn. *Dictyophimus gracilipes* Bailey, 1856 p. 4, pl. 1, Figure 8; Boltovskoy and riedel 1980, p. 124, pi. 5, Figure 8.
*Pseudodictyophimus gracilipes tetracanthus* Popofsky 1913; Leg 104. Leg 27 (p791 and Pl. 18);
Takahashi and Honjo 1981, p. 153, pI. 9, Figures 3 and 4.

This species was described earlier with the name *Dictyophimus gracilipes* by Bailey [57]. Later, this species was redescribed as *Pseudodictyophimus gracilipes tetracanthus* in 1971 by Petrushevskaya [54]. This species characterized by the spherical shape cephalic which contains numerous circular pores and large conical spines. The specific characters are the three divergent wing-like spines extended down-word from the thorax at the ventral side (Figure 8M). Previously, this species was reported from the Atlantic and the Pacific Ocean [17,39]. Recently, this species recorded for the first time at one station (St. I314) in the eastern Indian Ocean (Figure 1).

#### 3.11.6. *Cladoscenium ancoratum* Haeckel 1877 (Figure 8N)

The *Cladoscenium ancoratum* was originally described by Haeckel in 1877 in the central Pacific Ocean [39]. This species can be characterized by the campanulate shell contains the polygonal pores. The upper part of the cephalic portion has a proximal spine, which is further perforated into the three-blades or lateral branches. The basal plate has two large cardinal and two small jugular pores (Figure 8N). This is common Atlantic dweller species [17] which recorded for the first time at the one station (St. I314) in the eastern Indian Ocean (Figure 1).

#### 3.11.7. *Tetraphormis dodecaster* Takahashi 1991 (Figure 8O)

Syn. *Sethophormis dodecaster* Haeckel 1887, p 1248, pl. 56, Figure 12.
*Sethophormis* cf. *dodecaster* (HaeckeI) Takahashi and Honjo 1981, p. 152, pl. 8, Figure 8.

*Tetraphormis dodecaster* was originally described by Haeckel in 1862 in the South Pacific Ocean [39]. This species characterized by the compressed cephalic with primary or peripheral ribs, and eight end ribs interposed between the first and that occur some distance from the annular ring. Wrist with twelve protruding pointed lobes and twelve semicircular cavities between them (Figure 8O). The size of the cephalic measure as (0.081 mm); pores (medium to large; 0.0063–0.042 mm) and ribs (0.076 mm). This species recorded for the first time at the stations (Stas. I306, I807, I609) in the eastern Indian Ocean (Figure 1).

#### 3.11.8. *Tetraplecta pinigera* Haeckel 1881 (Figure 9A)

Syn: *Plectaniscus cortiniscus* Haeckel, 1887, p. 925, pI. 91, Figure 9.

*Tetraplecta pinigera* was described by Haeckel in 1881 in the Pacific Ocean [17]. This species has four pins shaped tree skeleton contain twelve delicate triangular wings and also rectangular meshes (Figure 9A). Three or four-bladed spines are straight or curved positions connected by the center (Figure 9A). Each spine has lateral branches associated with the delicate web-like structure (Figure 9A). This species recorded for the first time at the stations (Stas. I107, I308, I815) in the eastern Indian Ocean (Figure 1).  

#### 3.11.9. *Archibursa tripodiscus* Haeckel 1887 (Figure 9B)

*Archibursa tripodiscus* was described by Haeckel in 1887 in the Atlantic Ocean [6]. This species is characterized by the sub-spherical shell, with a smooth surface that has irregular pores. There are basal plates that have large and three triangle feet (Figure 9B). These three feet are widely divergent, straight, and three-sided prismatic as long as the diameter of the shell. This species was previously reported from the south Atlantic Ocean and the Pacific Ocean [17,39]. This species recorded for the first time at the station (St. I308; St. I413) in the eastern Indian Ocean (Figure 1).

#### 3.11.10. *Pterocanium korotnevi* Dogiel and Reshetnyak 1952 (Figure 9C)

Syn: *Lychnocanium korotnevi* in (Dogiel), Petrushevskaya and Kozlova, 1972, p. 553, PL 29, Figure 16.

*Pterocorys korotnevi* was described by Dogiel and Reshetnyak in the north Pacific Ocean [59,60,61]. This species can be characterized by the thorax with a cupola which perforated round, irregular-sized pores. Three massive, slightly convex (outward) base thorns extending from the lower edge of the cupola. The thorns extend from the edge of the cupola to each other at a 120° angle (Figure 9C). The pores on the surface of the cephalic are small and large on the thorax. This species was previously reported from the North Pacific Ocean [17,39]. This species recorded for the first time at the stations I107, I308, I310, I603 in the eastern Indian Ocean (Figure 1).

#### 3.11.11. *Pterocorys hertwigii* (Haeckel) Petrushevskaya 1971 (Figure 9G)

Syn. *Eucyrtidium hertwigii* Haeckel 1887; p. 1491 pl.80 Figure 12.
*Theoconus hertwigii* Nigrini, 1967, p. 73–74; pl. 7, Figure 4a,b.
*Pterocorys hertwigii* (Haeckel) in Caulet and Nigrini, 1988, p. 229–230; pl. 1, Figures 11 and 12.
*Pterocorys hertwigii* (Haeckel) Boltovskoy 1998, Figure 15.155.
*Pterocorys zancleus forma hertwigii* van de Paverd, 1995, p. 244; pl. 74, Figures 3 and 4.

This species was originally described by Haeckel in 1887 in the central Pacific Ocean [6]. Initially, this species has been known as *Eucyrtidium hertwigii* [6], and changed to new genus *Pterocorys* and the species is *Pterocorys hertwigii* which is still valid to date [11,62]. This species can be characterized by the conical or ovate shell, with three slight strictures, two apical spines (Figure 9G). The cap or cephalic is (0.026 mm) which has two apical spines (0.036 mm) and three-blade spines. Thorax is comparatively in a cylindrical shape which is about 0.061 mm, and three remarkable wings that further divided into two segments (t, ab, a) are situated. Pores are small in size (0.0028–0.0031 mm) which is equally distributed on the cephalic and abdominal surface. This species recorded for the first time at two stations (Stas. I404, 1406) in the eastern Indian Ocean (Figure 1).

#### 3.11.12. *Conarachnium parabolicum* (Popofsky) Takahashi 1991 (Figure 9H)

Syn. Sethoconus anthocyrtis Haeckel 1887, p. 1296, pl. 62, Figure 21.
*Lampromitra parabolica* Popofsky 1913, p. 348, Figure 54; Renz 1966, p. 122, pi. 4, Figure 14.

*Conarachnium parabolicum* was described by Haeckel in 1887 from sediment samples of the Atlantic and the Pacific Ocean [6]. Initially, this species was known as *Sethoconus anthocyrtis* which later changed into a new genus *Conarachnium* Popofsky [17]. The species has a small cephalic with additional accessory spines and a broader or wider thorax. The surface is smooth, delicate with hexagonal pores (Figure 9H). This species recorded for the first time at one station, namely (St. I605) in the eastern Indian Ocean (Figure 1).

#### 3.11.13. *Dictyocodon palladius* Haeckel 1887 (Figure 9I)

*Dictyocodon palladius* was described by Haeckel in 1887 from sediment samples in the central Pacific Ocean [6]. This species can be characterized by the small cephalic, with a large double-cone angle, which has small and usually branches like secondary spines at the base tip. Pyramid shape horns are on both sides and gradually expanding to the abdomen (Figure 9I). This is the first record of the species recorded from the stations (Stas. I406, I509, I817, I817, HF05) in the eastern Indian Ocean (Figure 1).

#### 3.11.14. *Eucecryphalus clinatus* Takahashi 1991 (Figure 9J)

*Euceryphalus clinatus* was described by Takahashi 1991 in the Atlantic Ocean and extended to the Pacific Ocean [17,61]. The shell is sub-spherical in outline, the cephalic and thorax are in sphere shape with the hexagonal pores, that arranged into 14 rows. The cephalic area has small pores and a short spine but the thorax has a smooth surface and beret shape with hexagonal pores. The pore area is 2 to 5 times broader than the gap bar (Figure 9J). This is the first record of this species at the stations (Stas. I101, I308) in the eastern Indian Ocean (Figure 1).

#### 3.11.15. *Eucyrtidium dictyopodium* (Haeckel) Takahashi, 1991 (Figure 9K)

Syn: *Stichopodium dictyopodium* Haeckel, 1887, p. 1447, pl. 75, Figure 6.

Haeckel in 1887 described the *Eucyritidium dictyopodium* in the Central Pacific Ocean [6]. Originally, this species has been known as *Stichopodium* but later, it was confirmed with the genus *Eucyritidium* which is valid to date [11]. The shell has a short cephalic and elongated or broader thorax within the six segmented abdomens in outline. The shell is 0.0022 mm with the small pores 0.0003–0.0005 mm and short apical spine 0.0004 mm. There are six portions on the abdomens segmented define as primary abdomen segment (Ps1) and post-abdomen segments (Ps2, Ps3, Ps4, Ps5, and Ps6). These segments are in the same sizes (0.0043 mm) and joint to each other with an internal septum. The last postabdominal segment is quite wider with an open mouth (Figure 9K) which can differentiate from the other *Eucyrtidium* species such as *E. hexansticum*. The species was distributed from the Atlantic Ocean and the Pacific Ocean [39]. This species recorded for the first time at the stations I509 and I807 in the eastern Indian Ocean (Figure 1).

#### 3.11.16. *Sethoconus venosus* Haeckel 1887 (Figure 10A,B)

Syn. *Phlebarachium venosum* Haeckel 1887, p. 1297, pl. 55, Figure 2.
Eucyritidinium venosum Takahashi, 1991.

*Senthoeonus vensous* was described by Haeckel in 1887 in the central Pacific Ocean and extends to the Atlantic Ocean [17]. This species was described as *Eucyritidinium venosum* by Takahashi [17]. This species has a very delicate shell (0.399 mm) in size, the cephalic is small than to thorax, the thorax is wider with three divergent radial beams. There are 4 club-shaped peristomial lobes (PLC). The surface meshes with polygonal pores which can be separated by bars (Figure 10A,B). Recently, this species recorded for the first time from the stations (Stas. I603, I611, I609) in the eastern Indian Ocean (Figure 1).

#### 3.11.17. *Theocorys veneris* Haeckel 1887 (Figure 10C)

Syn: *Theocorys creticum* Ehrenberg in Haeckel, 1887, p. 1415.
*Eucyrtidium veneris* van de Paverd 1995, p. 240; pl. 75, Figures 12–14.

*Theocorys veneris* was described as *T. creticum* by Ehrenberg 1857 and later this species was described by Haeckel in 1887 in the Pacific Ocean and the Atlantic Ocean [6]. This species can be characterized by the small cephalic which contains circular pores and long, conical apical spines. The shell is globular in shape, large thorax and abdomen have three-bladed spines that attached through the median bar. The pores are numerous, unequal on the surface (Figure 10C). This species recently reported from the South China Sea [63] as well as from the Mediterranean Sea [64]. This species recorded for the first time at the stations (Stas. I310, I413, I501, I509, I511, I601) in the eastern Indian Ocean (Figure 1).

#### 3.11.18. *Theopilium tricostatum* (Haeckel) Boltovskoy 1998 (Figure 10E)

Syn. *Theocalyptra sp.* Renz p. 137; pl. 5, Figure 13.
*Eucecryphalus tricostatus* Takahashi 1991, p. 110; pl. 33, Figures 4 and 6.
*Sethoconus tricostatus* van de Paverd 1995, p. 233; pl. 69, Figures 4–8.

*Theopilium tricostatum* was described by Haeckel 1887 from the surface sediment, south Atlantic, and the Pacific Ocean [17]. This species has common distribution in the Atlantic and Pacific Ocean [6,39,64,65]. Although this species has been reported as *Eucecryphalus tricostatus* from the Atlantic and the Pacific Ocean [8,63], and as *Sethoconus tricostatus* from the Netherland [64]. This species can be characterized by sub-spherical cephalic, campanulate thorax, and abdomen, with three-blade spines (Figure 10E). The cephalic is about (0.0031 mm), campanulate thorax is (0.0137 mm) and abdomen is (0.0063 mm) with numerous circular and polygonal pores. This species recorded for the first time at the stations (Stas. I310, I413, I501, I509, I511, I601) in the eastern Indian Ocean (Figure 1).

#### 3.11.19. *Cephalospyris cancellata* Haeckel 1887 (Figure 10F)

This species was originally described by Haeckel in 1887 from the surface sediment of the south Atlantic Ocean [6]. This species can be characterized by the reticulated shell with a network of polygonal pores and rudimentary horn (Figure 10F). The ovate shell has a long apical spine which is separated by the sagittal septum. There is a rudimentary horn placed at the frontal side. This species was reported in the south Atlantic Ocean [17,65]. This species recorded for the first time at the station 1817 in the eastern Indian Ocean (Figure 1).

### 3.12. Systematics and Morphology of Phylum Cercozoa-Pheodarian in the Eastern Indian Ocean

Cercozoa Cavalier-smith, 1998, emend. Adl et al. 2005, was erected to a new phylum based on its lack of distinctive morphological characteristics, such as many tubular cristae, and the cytoskeleton shell usually has filopodia and microbodies with extruosome [9]. It includes the order Phaeodarea Haeckel, 1879 (Tripylea) [66], which was characterized specifically by the perforation of one astrophile, two parapylae (Tripylae), and a phaeodium inside the extracapsular cytoplasmic area (Ext), enclosed by the scattered siliceous skeleton (Sc) and radial tubes around the shell [67,68]. On this morphological basis, 10 species were identified recently from the eastern Indian Ocean, and the following characteristics are described here.

### 3.13. Type Species of Pheodarian Species and Distribution in the Eastern Indian Ocean

#### 3.13.1. *Aulacantha scolymantha* Haeckel 1862 (Figure 11A)

**Figure 11:**
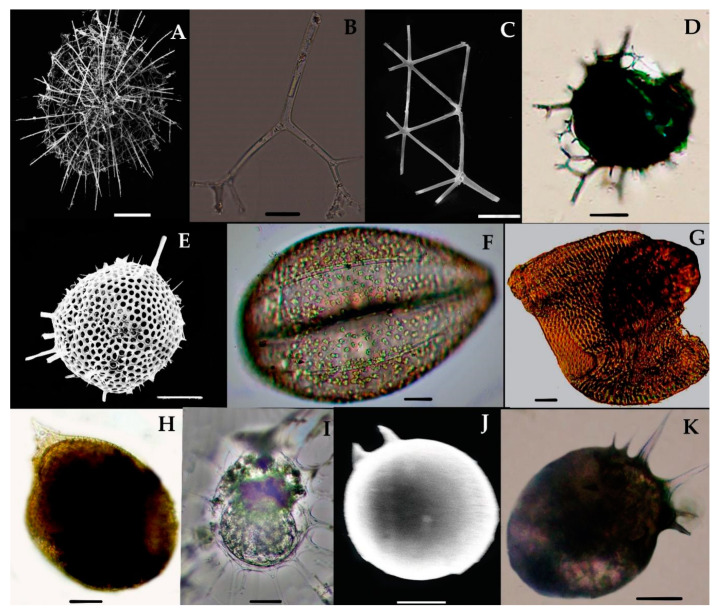
Photomicrographs of Phaeodaria species (**A**–**K**) of Eastern Indian Ocean. The images were modified from Munir et al. [27] (**A**) *Aulacantha scolymantha;* (**B**) *Auloceras arborescens subelegens*; (**C**) *Aulatractus ternaria*; (**D**) *Coelodendrum ramosissimum***; E**) *Castanidium longispiniim;* (**F**) *Conchidium compressa;* (**G**) *Conchidium capsula;* (**H**) *Conchidium caudatum;* (**I**) Pheodarian sp; (**J)** *Challengeron radians;* (**k**) *Pharyngella gastrula.* Scale bar: 10 µm, 100 µm.

*Aulacantha scolymantha* was described by Haeckel in 1862 and Hertwig in 1879 in surface sediments of the Pacific Ocean, the Atlantic Ocean, and the Indian Ocean [39,67]. Later, this species was also reported in the Mediterranean Sea [20]. The species can be described on the basis of its sclerocome shell enclosed by an external capsule. The intercapsulum is small and contains numerous needle or cylindrical radial tubes. These tubes have some dental teeth at the distal end (Figure 11A). This species was recorded for the first time at St. I817 in the eastern Indian Ocean (Figure 1).

#### 3.13.2. *Auloceros arborescens* subelegens Haeckel 1879 (Figure 11B)

Syn: *Auloceros arborescens birameus* (Immermann) Haecker, 1908b, p. 53, pI. 3, Figures 21–25, 34, 35;
pI. 10, Figure 102.

*Auloceros arborescens subelegens* was described by Haeckel in 1879; he identified this species from surface sediments of the Atlantic and Arctic Ocean [17]. The species can be described on the basis of radial tubes with 2 to 4 branches, with the cylindrical end at the distal point (Figure 11B). The whole tube is 497 µm, the length of the branches is 63–43 µm, and the circular end is 18 µm. This species occurs rarely in the eastern Indian Ocean and was recorded for the first time at the one station—namely, I308—in the eastern Indian Ocean (Figure 1).

#### 3.13.3. *Aularia ternaria* Haeckel (Figure 11C)

*Aularia ternaria* was described by Haeckel in 1862 in the north Pacific Ocean [39]. The shell has a thick, long, and triangular mesh of smooth tubes. There are six triangular nets, with seven nodal points connecting each tube (Figure 11C). The six triangular nets are 0.132 mm and the nodal pores are 0.09 mm. This species was reported in the southern part of the Atlantic Ocean and the Pacific Ocean [17]. This species was recorded for the first time at the stations I107, I509, and I815 in the eastern Indian Ocean (Figure 1).

#### 3.13.4. *Coelodendrum ramosissimum* Haeckel 1860 (Figure 11D)

*Coelodendrum ramosissimum* was described by Haeckel in 1860 [6]. The shell has hemispherical valves that are almost spherical in shape with Galea arches and a large nasal opening (Figure 11D). There are the four primary tubes which are further divided 4 to 6 times at a right angle (Figure 11D). These bifurcate branches are smooth, straight, slightly curved, and attached to the disc with about 4–7 short curved teeth. The shell size is 0.0017 mm, with divergent branches (0.003–0.0008 mm). This species is well distributed across the China Sea [69] and the eastern Mediterranean Sea [64]. However, this is the first record of the species found at a station (IQ) in the eastern Indian Ocean (Figure 1).

#### 3.13.5. *Castanella longispinium* (Haecker) Takahashi, 1991 (Figure 11E)

*Castanella longispinium* was described earlier by Haecker om the south Atlantic Ocean. The species has a spherical shell with numerous short spines and flame bars. The pores are circular and the bars are longer and thicker (Figure 11E). The pores are 0.0131 mm in size and the radial spines are 0.0159 mm. This species was reported in the Gulf of Oman [70] and the South Atlantic Ocean [26]. Recently, this species occurred for the first time at St. I503 in the eastern Indian Ocean (Figure 1).

#### 3.13.6. *Conchopsis Compressa* (Haeckel) Takahashi, 1991 (Figure 11F)

*Conchopsis compressa* was described earlier by Haeckel in 1887 in the north Pacific Ocean [6]. The shell is compressed or subcircular in outline, with a spindle-shaped cinctural perimeter (Figure 11F). The bivalve shell is 0.16 mm with small pores 0.0006 mm. The geographical range of this species is limited to the south Pacific Ocean [29]. This species was recorded for the first time and found at the stations I107, I505, I607, I609, and I611 in the eastern Indian Ocean (Figure 1).

#### 3.13.7. *Conchellium capsula* (Borgert) Takahashi 1991 (Figure 11G)

*Conchellium capsula* was described earlier by Borgert in the Pacific Ocean and in the Atlantic Ocean by [17,71]. This species has hemispherical valves without a sagittal keel nor projecting horns (Figure 11G). This species was recorded for the first time and found at the station HF05 in the eastern Indian Ocean (Figure 1).

#### 3.13.8. *Conchidium caudatum* Haeckel 1881 (Figure 11H)

*Conchidium caudatum* Haeckel in 1881 was originally described in the eastern Atlantic Ocean [39]. The shell is lenticular or compressed in outline, with an ovate sagittal and cinctural perimeter covered by the frontal elliptical and conical teeth (Figure 11H). The height of these conical teeth is 0.0171 mm. There are pores in the girdle fissure arranged irregularly in longitudinal rows which are separated by meridional ridges and converge to both ends of the main axis (Figure 11H). This species was recently recorded at one station (St. I507) in the eastern Indian Ocean (Figure 1).

#### 3.13.9. *Challengeron radians* Borgert 1904 (Figure 11J)

*Challengeron radians* was originally described by Haeckel in 1887 in the eastern Atlantic Ocean [6]. The species can be characterized by its amphora-shape structure, with 18–26 radial teeth equally spaced on the edges (Figure 11J). These marginal spines are conical and straight, and longer. These peristome teeth are large 0.018–0.0098 mm in a channel shape and are situated vertically (Figure 11J). This species was recorded at one station (St. I607) in the eastern Indian Ocean (Figure 1). The distribution of this species was reported before in the Pacific Ocean, the Atlantic Ocean [17], and the Japan Sea [72].

#### 3.13.10. *Pharyngella gastrula* Haeckel 1879 (Figure 11K)

Syn: *Protocystis thomsoni* (Murray) in Takahashi and Honjo,1981, p. 155 (partim), pI. 11, Figure 3.

*Pharyngella gastrula* Haeckel in 1879 was originally characterized and described in the central Pacific Ocean [73]. The species is characterized by its smooth and cylindrical or oval shaped shell with a long pharynx that has two teeth parallel to the peristome (Figure 11K). The pharynx descends from the outer and inner apertures. The size of the whole shell is 0.014 mm and the teeth are 0.99–0.004 mm. This species was recorded at one station—namely, St. I507—in the eastern Indian Ocean (Figure 1). The range of this species is from the southern part of the Atlantic Ocean to the Pacific Ocean [18,30].

## 4. Conclusions

In total, we identified 168 Radiolarian taxa, of which 60 species were described here for the first time from eastern Indian Ocean. These newly recorded species expanded their range from the eastern Indian Ocean and previously reported from the Mediterranean region and northwards to the Pacific Ocean, Atlantic Ocean This work provides the taxonomic information and distribution of the 60 Radiolarian species collected from the eastern Indian Ocean at a 200 m depth. The identified taxa were allocated into four groups from two phyla, Retaria (Acanthria, Taxopodida, Polycystine) and Cercozoa (Pheodarian), in the eastern Indian Ocean. Most of our material has a morphology slightly different to that of species described previously; the material comes from surface sediments found in the Haeckel report, so this is likely a new distribution discovered in the eastern Indian Ocean. For these morpho-species, the application of molecular procedures is required, which will lead to a more robust and precise taxonomic delimitation and will allow us to gain better knowledge of the diversity of radiolaria plankton in the eastern Indian Ocean. The classical taxonomical and molecular diversity of these planktonic organisms has still not been sufficiently investigated. Despite the ecological importance of these eukaryotic microbes in many oceanic areas, the phylogenetic position of these taxa has not yet been confidently established. This baseline study will be beneficial for future research and molecular analyses of the phylogenetic taxonomical classification of shell-forming Radiolarians.

## Figures and Tables

**Table 1:** Annotated list of Radiolarian species, classification, and newly recorded species from the eastern Indian Ocean.

Group-A	Group-B
Class Acantharia	Class Sticholonchea, (poche, 1913) petrushevskaja. 1977
Order Holacanthida	Order Taxopodida
Suborder Arthracanthida	Suborder sticholonchida schewiakoff, 1926
Family Acanthochiasmidae Haeckel, 1862	Family Sticholonchida Hertwig, 1887
* *Acanthochiasma fusiforme*, * *Trizona brandti*	Genus sticholonche hertwig, 1887
Family Phyllostauridae Schewiakoff, 1926	* *Sticholonche zanclea*
* *Acanthostaurus conacanthus*	Group-D
Family Dictyacanthidae Schewiakoff, 1926	Order Nassellaria
* *Dictyacantha tabulate*	Suborder Collodaria
* Tessaropelmida	Family Collozoidae Haeckel, 1862
Suborder Symphyacanthida	Subfamily Collospheroidea Muller, 1858
Family Amphilithidae Haeckel, 1887	Solenosphaera zanguebarica, Collosphaera huxleyi, Collosphaera macropora, Collosphaera tuberosa, Disolenia quadrata, Siphonosphaera polysiphonia, Siphonosphaera socialis, * Siphonosphaera magnisphera * Collozoum inerme
* *Amphilonche concreta*	Family Thalssopheroidae Haeckel, 1862
Suborder Sphaenacantha	* *Thalassoxanthium cervicorne,*
FamilyDiploconidae Haeckel, 1887	* *Thalassoxanthium octoceras*
* *Diploconus saturnus*	Family Artostrobiidae Riedel, 1967 emend. Foreman, 1973
Group-C	*Botryostrobus auritus-australis, Spirocyrtis subscalaris*
Phylum CERCOZOA Cavalier-Smith, 1998	Family Carpocaniidae Haeckel, 1881 emend. Riedel, 1967
Class Thecofilosea t. Cavalier-Smith and E.E.-Y. Chao, 2003	*Carpocanium* sp., *Acrosphaera spinosa*/*cyrtodon*,
Subclass Phaeodaria Haeckel, 1879	*Euchitonia furcata, Euchitonia triangulum, Spongodiscus craticulatus, Spongodiscus resurgens, Spongotrochus glacialis, Spongaster tetras, Spongurus cylindricus, Spongurus pylomaticus, Stylodictya aculeata, Stylodictya multispina,*
Order Phaeosphaerida Haeckel, 1887	Stylochlamydium asteriscuss, Buccinosphaera invaginata
Family Aulacanthidae Haeckel, 1862	Suborder Nassellaria incertae sedis ()
* *Aulacantha scolymantha,*	Super family Eucyrtidiacea
* *Auloceros arborescens subelegens*	Family Theoperidae Haeckel, 1881 Emend. Riedel, 1967
Family Aulosphaeridae or Medusettidae Haeckel, 1887	*Conarachnium parabolicum, Corocalyptra cervu, Corocalyptra elisabethea, Corocalyptra craspedota, Cycladophora davisiana, Cycladophora rosette, Dictyophimus macropterus, Dictyocodon palladius, Eucecryphalus clinatu, Eucecryphalus gegenbauri,*
* *Aularia ternaria, Alusophaera trigonopa*	*Eucecryphalus sestrodiscus, Eucecryphalus craspedota, Eucytridium cienkowiski, Eucyrtidium venosum, Eucytidium hexastichum, Eucytidium dictyopodium, Litharachnium tentorium, Lipmanella bombus, Theocorys veneri, Theopilium tricostatum, Sethoconus anthocyrtis.*
Family Coelodendridae Haeckel, 1887	Family Plagiacanthidae Hertwig, 1879
* Coelodendrum ramosissimum	*Arachnocorallium calvata, Cystidium princeps,*
Family Castanellida Haeckel, 1879	*Callimitra emmae,*
* *Castanella longispinium*	*Callimitra carolotae, Clathrocanium coarctatum,*
Order Phaeoconchia Haeckel, 1879	*Clathrocorys teuscheri, Clathrocanium murrayi,*
Family Conchariidae Haeckel, 1879	*Ceratocyrtis histricosus, Cladoscenium aucoratum,*
* *Conchopsis compressa,*	*Cladoscenium tricolpium, Cladoscenium limbatum,*
* *Conchelliumcapsula,*	*Lophospyris pentagona pentagone, Lophophaena hispida-cylindrica, Lophophaena dodecantha.*
* *Conchidium caudatum,*	Family Pterocorythidae Haeckel, 1881
* *Challengeron radians*	*Archibursa tripodiscus, Anthocyrtidium sp.,*
Family Challengeridae Murray, 1885	*Anthocyrtidium cineraria, Anthocyritidium ophirens,*
* *Pharyngella gastrula*	*Lamprocyclas maritalis, Pterocorys campanula,*
Group-D	*Pterocorys zancleus, Pterocanium korotnev,*
Class Polycystinea Ehrenberg, 1838	*Pterocanium hrilolum, Pterocanium pretextum,*
Order Spumellaria	*Theocorythium trachelium, Theocorythium tricostas,*
Family Actinommidae Haeckel, 1862, Emend. Riedel, 1967	*Stichopilium bicorne.*
Subfamily Astrosphaeridae Haeckel, 1881	Family Trissocylidae Haeckel, 1881, Emend. Goll, 1968
*Acanthosphaera actinota, Acanthosphaera dodecastyla, Actinomma arcadophorum, Actinosphaera capillaceum, Acanthosphaera pinchuda, Actinosphaera tenella, Arachnosphaera myriacantha, Astrosphaera hexagonalis, Centrocubus cladostylus*	*Amphispyris reticulata, Cephalospyris cancellata,*
*Cromyechinus circumtextum, Cladococcus scoparius, Cladococcus cervicornis, Cladococcus megaceros, Cladoccocus abietinus, Drymosphaera* cf. *polygonalis*	*Ceratospyris hyperborea, Ceratospyris mulderi,*
Subfamily Ethmosphaeridae	*Dictyospyris sp., Eucecryphalus craspedota,*
*Elatomma penicillus, Hexalonche amphisiphon, Heliosphaera radiata,*	*Eucytridium cienkowiski, Eucyrtidium venosum,*
*Gonosphaera primordialis, Cyrtidosphaera reticulata, Hexacontium armathostile, Hexastylus, Haeckeliella macrodora, Octodendron cubocentrum, Spongosphaera streptacantha, Spongodictyon*	*Eucytidium hexastichum, Eucytidium dictyopodium, Litharachnium tentorium, Lipmanella bombus, Theocorys veneris, Theopilium tricostatum, Tholospira cervicornis*
*spongiosum,* * *Stylosphaera spongiacea*	*Sethoconus anthocyrtis, Semantis gracilis, Zygocircus productus*
*Spongodrymus elaphococcus, Xiphosphaera tessaractis, Xiphosphaera*	
Family Coccodiscidae Haeckel, 1862	
*Didymocyrtis tetrathalamus*	
Family Litheliidae Haeckel, 1862	
*Larcopyle buetschlii*	
Family Pyloniidae Haeckel, 1881	
*Octopyle stenozona/Tetrapyle octacantha,*	
*Streblacantha circumtexta*	
Family Spongodiscidae Haeckel, 1862 Emend. Riedel, 1967	
*Amphirhopalum ypsilon, Dictyocoryne profunda,*	
*Dictyocoryne trucatum, Dictyocodon palladius,*	
*Dictyocoryne euclidis, Euchitonia elegans,*	

Note * indicate the first record of the species in the eastern Indian Ocean.

**Table 2:** Systematic notes and morphometric measurements of the newly recorded species of the eastern Indian Ocean.

Radiolarian Groups		Species	Shell Outline	Central Capsule (Int)/Medullary Shell	External Capsulum (Ext)/Cortix Shell	Cephalis (ce)	Thorax (th)	Abdomen (ab)	PL (Protoplasmic Spine)	Texture	Pores	Spines	Basal Feet (bf)
Acanthria		*Acanthochiasma fusiforme*	Star-shape	Small, spherical (0.035 mm)	Colorful	None	None	None	None	Porous	Small	10–20 dimeteril radial, spindle spines (0.57 mm)	None
		*Trizona brandti*	Star-shape	Small, spherical (0.16 mm)	Colorful	None	None	None	None	Porous	Medium	Radial, needle shape, 10–20 diameter (0.57 mm)	None
		*Acanthostratus conacanthus*	Star shape	Small, spherical (0.25 mm)	Colored (0.16 mm)	None	None	None	None	Porous	Medium	Radial, blunt shape, small 10 diameter (0.57 mm)	None
		*Dictyacantha tabulata*	Spherical shell with polygonal meshes	Intercapsulum (0.16 mm)	Mesh type, transparent (0.26 mm)	None	None	None	None	polygonal meshes	Rectangular pores	Thin spines (0.18 mm)	None
		*Amphilonche concreta*/*Amphilonche elongata*	Elongated shell, has rod type spines at the edges	Smooth	Broken	None	None	None	None	Smooth	Invisible	Bi-spines,short (0.01 mm)	None
		*Diploconus cylindrus*	Large shell has cylindrical or conical shape cornet and ribs	Spherical, small (0.003 mm)	Wider and fan shaped (0.0084 mm)	None	None	None	None	Smooth attached with ribs and horns (0.074 mm)	Microperforate, ribs	Shorts (0.0084 mm)	None
		*Diploconus faces*	Bell-shaped shell	Small, spherical (0.019 mm)	Small	None	None	None	None	Smooth, porous with ribs and horns	Medium	Pin-like curved spicules	None
Taxopodia		*Sticholonche zanclea*	Capsular cell with Oar-like axopods spines	Capsular cell (0.24 mm)	None	None	None	None	None	Smooth	None	Oar-like axopodia (0.44 mm)	None
Pheodaria		*Aulacantha scolymantha*	Lattice shell, has cylinderial radial tubes and well developed sclerocome	Intercapsulum are short with numerous tadial tubes.	Circular with capsular wall	None	None	None	None	Smooth	None	Radial tubes with teeth-like dentation at the distral end	None
		*Auloceras arborescens*	Radial tubes, has 2 to 4 branches, are circular at the distal end.	None	None	None	None	None	None	Smooth and branching	None	2–4 branches, cylinderical at the tip	None
		*Aulatractus ternaria*	Thick, has smooth, long triangular mesh tubes.	None	None	None	None	None	None	Six triangular nets, with seven nodal points of tubes (0.132 mm)	Nodal pores (0.09 mm)	None, no radial tubes	None
		*Coelodendrum ramossissimum*	Round central capsulum has divergent branches at the radial spine.	Dark colored (0.0017 mm)	Pentagonal meshes	None	None	None	None	Two thin-walled hemispherical valves, has conical gaea which again has more divergent branches	None	Divergent branches (0.003–0.0008 mm)	None
		*Castanidium longispiniim*	Lattice spherical shell, has numerous short spines and bars.	Large, spherical	Large and porous (0.0131 mm)	None	None	None	None	rugose, and porous	round	radial spines short with thick flame (0.0159 mm)	None
		*Concliellium capsula*	Lattice bivalve shell, lobulate, has elongate chambers. with horn on aboral hinge.	Trochospiral	Left and right	None	None	None	None	Smooth to hispid	Medium to large		None
		*Conchidium compressa*	Compressed or subcircular shell, with spindle shape cinctural perimeter	Bivalve (0.16 mm)	Spindle shaped	None	None	None	None	Spiny	Small pores, (0.0006 mm)	None	None
		*Conchidium caudatum*	Lenticulate, compressed, ovate sagittal. Cinctural perimeter and frontal elliptical and conical Teeth.	Conical, compressed	Colored and porous	None	None	None	None	Smooth	microperforated (0.0009 mm)	Teeth-like (0.0171 mm)	None
		*Challengeron radians*	Amphora-shaped structure, with 18-26 equally spaced, radial teeth on the margin.	Compressed (0.046 mm)	Left and right	None	None	None	None	Lens-shaped, compressed	Microperforated	Spines on sharp marginal edge of shell, pointed teeth are longer than marginal spine (0.018 mm0.0098 mm)	None
		*Pharyngella gastrula*	Ovoid and bilaterical symmetrical shell, has peristome and two parallel and pointed teeth.	Non lattice shell, with pyranx and mouth	None	None	None	None	None	Ovoid, smooth surface	None	Two pointed teeth (0.0049–0.0047 mm)	None
Collodaria		*Siphonosphaera magnisphaera*	Sphere and non-lattice shell, has polygonal surface, without spines.	None	None	None	None	None	None	Rougose	4 large pores, numerous small (0.0017–0.0003 mm)	None	None
		*Sphaeozoum punctatum*	Colonies, non-lattice shell and spherical has spicules.	Sphere with central vacuole; (0.22 mm)	Sphere with capsular wall	None	None	None	None	Smooth with symbionant parasite	None	Spicules, rod shape with three needle spines; (0.096 mm)	None
		*Sphaerozoum fusum*	Colonies, spherical in shape	Contian the central vacuole	Contains symbiotic parasite’ (Sy) (0.021 mm)	None	None	None	None	Smooth and spiny spicules	None	Spicule rod has long, spiny needle; rod (0.03 mm); lateral branches (0.0003 mm), length of spicule (0.132 mm)	None
		*Sphaerozoum ovodimare*	Spherical, has loosely attached spicules	Colored (0.216 mm)	Colored with celleform bodies	None	None	None	None	Smooth	None	Three spicules shrank are shorter than middle rod, loosely detached to the skeleton (0.0315 mm)	None
		*Collozoum inerme*	Spherical, colored cytoplasm, without spicules	(0.239 mm)	Colored with celleform bodies	None	None	None	None	Smooth	None	Symbionants, no spicules (0.025 mm)	None
		*Thalassoxanthium cervicorne*	Siliceous spicules has three triangles equally spaced shanks, has three or more branches on each distal end.	None	None	None	None	None	None	Smooth	None	Three triangles shanks with branches (0.234 mm) and branches (0.0086–0.00152 mm)	None
		*Thalassoxanthium octoceras*	Spicules contains a short rod which has four diverging shanks	None	None	None	None	None	None	Smooth	None	Short intermediate rod (0.0075 mm) and four diverging shanks (0.109–0.1199 mm)	None
Spumellaria		*Acanthosphaera actinota*	Lattice shell, lobulate to compressed.	Porous	Thin meshes on cortix shell	None	None	None	None	Smooth to hispid	Large polygonal pores	Thread like bars (6–8) or radial ridges	None
		*Acanthosphaera dodecastyla*	Lattice shell, Larger, has circular pores and three bladed spines	Spherical	Right	None	None	None	None	Smooth to hispid	Large	Three-bladed spines	None
		*Acanthosphaera pinchuda*	Lattice shell has circular pores contains thread like spines	None	Sphere	None	None	None	None	Rugose	Large and circular	Thread like spines at the noda points	None
		*Actinosphaera capillacea*	Spherical and lattice shell has thin bars and circular pores	Medullary shell (0.043 mm)	Thin polygonal meshes (0.0149 mm)	None	None	None	None	Smooth	Circular, medium (0.00030 mm)	Bar-like spines and three bladed spine, thin (0.024 mm)	None
		*Actinosphaera tenella*	Concentric shell, oval and spherical	Medullary shell is porous	Cortix shell with large pores	None	None	None	None	Smooth to hispid, crystalline	Circular and Large	Single radial bar and numerous bristle. Radial spine (0.12 mm0 and bristle spines (0.1002 mm)	None
		*Arachnosphaera myriacantha*	Concentric and sphere-shape shell	Sphere with regular hexagonal meshes		None	None	None	None	Smooth network		Branches like cobweb network and spines	None
		*Centrocubus cladostylus*	Non-discoidal shell, contains 8 radial spines and spongy mesh network	(0.0030 mm)	Left	None	None	None	None	Meshnetwork	Large, polygonalnet (0.0018 mm)	Radial spines more than 6 (0.012 mm)	None
		*Cromyechinus circumtextum*	Concentric shell, have polygonal meshes at the cortix shell.	Spherical	Polygonal meshes	None	None	None	None	Smooth to network frame	Large	Three bladed spines and polygonal meshes having short bars.	None
		*Elatomma penicillus*	Disc-shaped biconvex	Trochospiral	Left	None	None	None	None	Smooth to hispid	Medium	None	None
		*Hexalonche amphisiphon*	Lattice shell with thin wall, has bristle-like radial spines	(0.523 mm)	Octahedral medullary shell has polygonal meshes	None	None	None	None	Smooth network	Hexagonal pores on cortix, polygonal pores on medullary shell (0.039 mm)	Six main radial spines (0.27 mm)	None
		*Styptosphaera spongiacea*	Concentric sphere shell with spongy framework	Small and compact	Extercapsulum, cortix sponge type (0.068 mm)	None	None	None	None	Smooth to spongy network	Medium (0.046 mm)	None	None
		*Spongurus pylomaticus*	Ellipsoidal shell has dense spongy meshes at the surface and bristle spines at the apex point.	Dense spongy	Looser meshwork	None	None	None	None	Smooth to hispid	Small, circular	Bristle shaped spines at the edges	None
		*Xiphosphaera tessaractis*	Single, lattice-sphere has two or more spines	Sphere and porous (0.12 mm)	None	None	None	None	None	Smooth to hispid	Small, circular (0.013 mm)	Three radial spines (0.027 mm)	None
		*Streblacantha circumtexta*	Symmetrical shell, has unequal pores and needle like spines at the boundary of cell.	Spherical	None	None	None	None	None	Rugose	Medium to large, unequal	Needle-like spines	None
		*Stylodictya longispinus*	Spherical, ring disc, wheel-shaped	Inner shell (11.11 um)	Rings are shorter as 8 or 12	None	None	None	None	Smooth and porous	Circular pores, small to medium (4.3 um)	Radial spines, thin (189.2 mm)	None
		*Stylochlamydium venustum*	Concentric and disk shape shell		Spongyform	None	None	None	None	Smooth and porous	Small to medium (0.0041 mm- 0.0061 mm)	Numerous radial spines (0.134 mm)	None
Nassellaria		*Cystidium princeps*	Large central capsules, ovoid in shape, with simple circular Porohora surrounded by the red pigment granules	Ovoid	Circular	None	None	None	None	Smooth	None		None
		*Callimitra carolotae*	Subspherical cephalic, thorax pyramidal has mesh networks and three vertical wings.	None	None	Subspherical	Pyramidal (0.0819 mm)	Mesh network	None	Smooth	Microperforated	Long spiny (0.076 mm)	None
		*Lophophaena capito*	Spherical and domp shaped shell	None	None	Bulb shaped (0.120 mm), with spines	Smooth and cylindrical	Spherical and porous	Invisible	Smooth to hispid	Circular, polygonal pores	Three bladed spines (0.075 mm)	Three bladed
		*Lampromitra schultzei*	Large cephalic, ragged to compressed shape thorax wih large pores surface.	None	None	Domp shape (0.039 mm)	Large, Mesh surface (0.128 mm)	Invisible	Invisible	Smooth and porous	rectangular pores (0.023 mm– 0.055 mm)	(0.12 mm)	None
		*Plectacantha trichoides*	Cephalic has loose network of arches,large and irregular pores	None	None	Small	Loose network	Invisible	None	Network of arches	Large and irregular	Short	None
		*Pseudodictyophimus gracilipes*	Cephalis has three divergent spines extending obliquely downward from the thorax and abdomen	None	None	Spherical with circular pores	Porous	Porous	Invisible	Porous	Circular	Large conical apical spine (s)	Three divergent basal feets
		*Tetraplecta pinigera*	Three-bladed spines joint at the mid-point of the cell, delicate branches emerging out from the each spines	None	None	Small	None	None	None		None	Lateral spines	Three to four basal feet
		*Tetraphormis dodecaster*	Shell flattened, has small cephalis, and conical thorax, contains twelve prominent nerves.	None	None	Small, spherical (0.081 mm)	Conical, porous (0.068 mm)	None	Invisible	Smooth and porous	Medium to large (0.0063–0.042 mm)	Ribs (0.076 mm)	Branches as nerves
		*Archibursa tripodiscus*	Shell subspherical, smooth with three basal feet and triangular pores, basal view	None	None	Invisible	Conical	Invisible	Invisible	Smooth and porous	three triangular pores	Invisible	Three feet widely divergent, straight, three sided prismatic,
		*Pterocanium korotnevi*	Shell is dome shape cephalis with cupola thorax and three basal feet spines	None	None	Sphere, domp shaped spines	Cupola type and porous	Spherical	Invisible		porous	Apical spine	Three basal feet curved
		*Pterocorys hertwigii*	Shell conical, with three slight strictures.	None	None	Large, conical, with two apical spines (0.0267 mm)	Cylinder with three remarkable wings and divided into two segments (0.061 mm)	Two segments (0.071 mm)	Invisible	Smooth and porous	Small, circular (0.0028–0.0031 mm)	Conical spines (0.036 mm)	Three bladed
		*Conarachnium parabolicum*	Shell conical, with small cephalis and broader thorax	None	None	Small with spines	Wider and broader	Invisible	Invisible	Smooth, cone shaped	Hexagonal	Short apical and accessory cephalis spines	Invisible
		*Dictyocodon palladius*	Shell with distinct collar have evanescent lumbar stricture	None	None	Short	Thorax three-sided pyramidal	Inflated, gradually dilated abdomen	Invisible	Smooth and porous	Equal and circular	Large pyramidal horn	
		*Eucecryphalus clinatus*	Subspherical shell and small cephalis and thorax with hexagonal pores	None	None	Spherical and small	Thorax is a smooth and unique beret- shaped		Invisible	Smooth and porous	Hexagonal pores	Invisible	None
		*Eucyrtidium dictyopodium*	Short cephalis, thorax with six abdomen sections	None	None	Subspherical (0.0022 mm)	Cylindrical and small (0.0037 mm)	Wide, porous, six segmented	2 PL lobes	Smooth and porous	Small (0.0003–0.0005 mm)	Short (0.0004 mm)	(0.0043 mm)
		*Sethoconus venosum*	Spherical shell with cephalis and thorax, supported by three divergen radial beams and network of mesh polygonal pores	Central capsule with 4 PLC		Subspherical (0.399 mm)	Wider and with mesh network		4 PL lobes	Delicate network of polygonal meshes	Polygonal meshes	Radial beams	Invisible
		*Theocorys veneris*	Small cephalis and large thorax with three bladded spines	None	None	Short (0.0025 mm)	Thorax spherical, large (0.0053 mm)	Short	None	Smooth and porous	Small (0.0004 mm)	Conical apical horn (0.0034 mm)	Two basal feet, (0.0064 mm)
		*Theopilium tricostatum*	Spherical, non-lattice shell	None	None	Subspherical (0.0031 mm)	Campulate thorax (0.0137 mm)	Spherical abdomen (0.0063 mm)	Three (0.0039 mm)	Smooth and porous	Circular, polygonal pores (0.0021 mm)	Apical spine (0.028 mm)	None
		*Cephalospyris cancellata*	Ovate shell, deep sagittal stricture with complete ring. Delicate meshes, irregular polygonal pore, rudimentary horn at apex.	Deep sagittal stricture	Mesh type and delicate	has horns	Deep sagittal stricture	None	None	Smooth and mesh type	Polygonal pores	Apical spines and rudimentary horns	Three basal feet curved

## Data Availability

Not applicable.
